# Researcher engagement in policy deemed societally beneficial yet unrewarded

**DOI:** 10.1002/fee.2084

**Published:** 2019-07-30

**Authors:** Gerald G Singh, Vinicius F Farjalla, Bing Chen, Andrew E Pelling, Elvan Ceyhan, Martin Dominik, Eva Alisic, Jeremy Kerr, Noelle E Selin, Ghada Bassioni, Elena Bennett, Andrew H Kemp, Kai MA Chan

**Affiliations:** ^1^ Nereus Program, Institute for the Oceans and Fisheries University of British Columbia Vancouver Canada; ^2^ Institute for Resources, Environment, and Sustainability University of British Columbia Vancouver Canada; ^3^ Department of Ecology Institute of Biology Universidade Federal do Rio de Janeiro Rio de Janeiro Brazil; ^4^ Northern Region Persistent Organic Pollution Control Laboratory Faculty of Engineering and Applied Science Memorial University of Newfoundland St John's Canada; ^5^ Department of Physics University of Ottawa Ottawa Canada; ^6^ Department of Biology University of Ottawa Ottawa Canada; ^7^ Institute for Science, Society and Policy University of Ottawa Ottawa Canada; ^8^ Department of Statistics University of Pittsburgh Pittsburgh PA; ^9^ SUPA, Centre for Exoplanet Science School of Physics & Astronomy University of St Andrews St Andrews UK; ^10^ Trauma Recovery Lab, MUARC Monash University Melbourne Australia; ^11^ Institute for Data, Systems, and Society and Department of Earth, Atmospheric, and Planetary Sciences Massachusetts Institute of Technology Cambridge MA; ^12^ Chemistry Department Faculty of Engineering Ain Shams University Cairo Egypt; ^13^ Department of Natural Resource Sciences and McGill School of Environment McGill University St Anne‐de‐Bellevue Canada; ^14^ Department of Psychology and Health and Wellbeing Academy College of Human and Health Sciences Swansea University Swansea UK

## Abstract

Maintaining the continued flow of benefits from science, as well as societal support for science, requires sustained engagement between the research community and the general public. On the basis of data from an international survey of 1092 participants (634 established researchers and 458 students) in 55 countries and 315 research institutions, we found that institutional recognition of engagement activities is perceived to be undervalued relative to the societal benefit of those activities. Many researchers report that their institutions do not reward engagement activities despite institutions’ mission statements promoting such engagement. Furthermore, institutions that actually measure engagement activities do so only to a limited extent. Most researchers are strongly motivated to engage with the public for selfless reasons, which suggests that incentives focused on monetary benefits or career progress may not align with researchers’ values. If institutions encourage researchers’ engagement activities in a more appropriate way – by moving beyond incentives – they might better achieve their institutional missions and bolster the crucial contributions of researchers to society.

Scientists have much to offer society, including the directbenefits of research and technology, increased public understanding of science and policy, informed democracy, and science‐based policy. Realizing these benefits often requires that researchers engage beyond academic communities, but this depends in part on institutional support (Hauser and Katz 1998; Franceschini *et al*. 2014). Scientific institutions often proclaim engagement to be a public good, but institutional values, strategies, and actions may dissuade researchers from participating in the very activities that provide important public benefits (Hauser and Katz 1998; Brembs *et al*. 2013). This is true even though public support for science has always been linked to the immediate or eventual benefits it provides (Sarewitz and Pielke 2007; Baron 2016).

Researchers’ activities are often grouped into four broad categories: research, teaching, service (eg sitting on committees), and policy and public engagement (Lach *et al*. 2003; Singh *et al*. 2014). Engagement – generally defined as collaboration between research institutions and surrounding communities for the mutually beneficial exchange of knowledge and resources in a context of partnership and reciprocity (Leshner 2003; Driscoll 2008) – is broadly viewed as an important activity to be encouraged (Singh *et al*. 2014). Many universities developed engagement programs in the 1980s as a way of defending their own public relevance by ensuring academic involvement in societal progress and innovation (Holland 2016). Many research and scientific institutions include societal and policy engagement in their mission statements, yet previous research indicates that mission statements alone – without consistent institutional support in the form of funding and reward structures – are not enough to foster engagement (Bernardo *et al*. 2012; Fitzgerald *et al*. 2016; Holland 2016).

Whereas research and teaching can be evaluated with relatively well‐developed – albeit controversial – metrics and processes (eg impact factors, UK's Research Assessment Exercise, teaching evaluations by students and peers), the ways to evaluate engagement are, at best, nascent (Brembs *et al*. 2013; Baron 2016). A key complication for such metrics is that excellence in engagement is multifaceted (Taylor 2007). We recognize the presence of seven “dimensions” that can be used to evaluate a researcher's engagement efforts (Franz *et al*. 2012). These dimensions include *reach* (the size of the audience), *rigor* (how research‐based the engagement is), *innovation* (novelty of engagement), *number* (quantity of effort), *depth* (amount of work behind each effort), *prominence* (perceived esteem of the effort), and *outcomes* (changes resulting from the effort; Table 1).

**Table 1:** Dimensions of engagement explored in this study, with definitions

Dimension	Definition or example
Reach	Size of audience, readership, etc
Rigor	Quality of representation of science
Innovation	Novelty of the engagement activity
Number/quantity of effort	Number of times engagement activities occur
Depth of effort	Magnitude of work and expertise behind each effort (eg writing op‐eds > signing petitions)
Prominence	Perceived esteem (eg keynote talks > invited talks > uninvited talks)
Outcomes	Intended or positive changes as a result of the activity

When setting goals for a particular engagement activity, it is important to determine when and how those goals will be considered to have been met (eg metrics of success). This requires a thorough understanding of the existing beliefs and values surrounding the activity being targeted. For example, when designing systems to evaluate and reward engagement, it is important to consider what researchers think about engagement, what motivates them to engage, and how they believe such activities benefit society. Although perceptions may differ from reality, they are important because they serve as the foundation for behavior (Jones and Nisbett 1971; Lerner *et al*. 2015). Perceptions are also key for determining whether individual and institutional goals align with each other, and with evaluation metrics. Previous research indicates that individuals’ public engagement efforts are more effective when they feel that their institution shares their values and supports their efforts (Jin *et al*. 2016). Researchers may become apathetic or cynical when they are incentivized to perform activities they see as having little value, or when institutional rhetoric that promotes such activities is not supported by evaluation metrics that place value on them (Colvin and Boswell 2007). Alternatively, participation in certain activities with perceived societal benefit (ie those that contribute to a better world) that are not measured or valued by their institution may jeopardize scientists’ careers. In this study, we investigated how researchers perceive the social importance of various engagement efforts and how institutional rewards encourage these activities, with a focus on the evaluation of and motivation for engagement activities.

We conducted an international survey of established researchers (defined below) and students to capture the views of both current and emerging researchers. If evaluation metrics aligned with institutional rhetoric regarding social benefit, we would expect to see a close correlation between perceived societal benefit of and perceived reward for various endeavors (ie research, teaching, service, and engagement). We hypothesized further that engagement activities are evaluated on an ad hoc basis and are considered narrowly (relative to the multiple dimensions of engagement excellence we identified). Finally, we expected that different motivations (including self‐oriented ones, such as career benefits, and selfless ones, such as combatting poor policy) would be associated with different engagement activities.

## Methods

We developed a survey questionnaire (WebPanel 1) and made it available online to “established researchers” (university, government, NGO, and industry staff with a PhD) and students (including postdocs) around the world. The survey was disseminated over researcher listservs (including ECOLOG‐L and the listserv for the Society for Conservation Biology [SCB]), as well as through the Global Young Academy and the Leopold Leadership Fellows organization, and recipients were asked to forward the survey to their colleague networks (see WebPanel 2 for how we tested for bias, given this sampling). Systematic sampling was also performed, with invitations to both participate in the survey and distribute it to heads and deans of research organizations.

In total, 634 established researchers and 458 students (overwhelmingly graduate students, with a few undergraduate students and some postdoctoral researchers) from 315 institutions and 55 countries participated in the survey (WebTable 3). Most respondents were natural scientists (51% of researchers, 39% of students) and interdisciplinary scientists (10% of both researchers and students; 17% of researchers and 36% of students did not provide information about discipline) from academic institutions in North America (53% of researchers, 50% of students), although there was also relatively strong representation from several other countries (eg Australia, Brazil, Japan, South Africa, Turkey, and the UK; WebTable 3). Although the survey was open to any researcher from any discipline or organization, the dissemination of our survey relied in part on subscribers to the SCB and ECOLOG‐L listservs. Therefore, ecologists and conservation scientists made up a large proportion of our sample.

**Figure 1:**
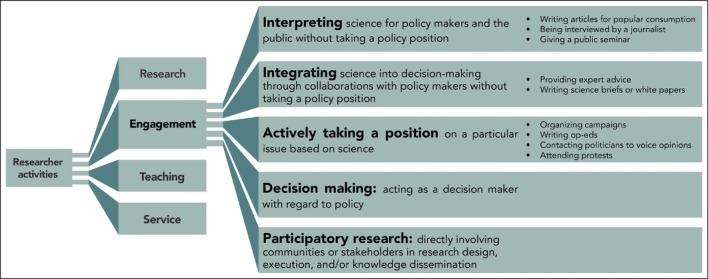
The five categories of engagement in this study. Engagement is one of four types of activities (along with research, teaching, and service) that researchers perform.

Our questions focused on the institutional metrics and perceived level of reward and societal benefit for various activities (research, teaching, service, and engagement), as well as how engagement is evaluated. We further broke down engagement into five categories (partially adapted from Singh *et al*. 2014): (1) interpreting science for policy makers and the public without taking a policy position; (2) integrating science into decision making without taking a policy position; (3) actively taking a position on a particular issue based on science; (4) acting as a decision maker with regard to policy; and (5) directly involving communities or stakeholders in research design, execution, and/or knowledge dissemination (Figure 1). We used Likert scales to quantify directional categorical responses to questions about societal benefits, institutional rewards, and quantity of engagement, as well as levels of agreement to statements about researcher motivations to engage. We concluded the survey by asking respondents if they would prefer that their institutions consider different metrics for each category of engagement, to place higher emphasis on rewards and expectations, and/or to not reward the activity at all. All demographic information was collected at the end of the survey.

We used colored matrices and bar plots to visualize data on perceptions and motivations, ANOVA followed by Tukey tests on pairwise contrasts to explore differences in societal benefits and institutional rewards for activities, and model averaging to study the relationships between stated motivations to engage and levels of engagement behavior (ie how often experts actually contributed to different engagement activities). Results were largely consistent across disciplines and geographic regions, but some differences were found between established researchers and students, and between early‐career and late‐career researchers; these differences are explored in more detail below (see WebPanel 2 for a description of the model averaging methods, considerations with respect to sampling bias, and geographical and disciplinary comparisons). The statistical software R (R Core Team 2013) was used for all analyses.

## Results and discussion

### Engagement is valuable but garners little reward

**Figure 2:**
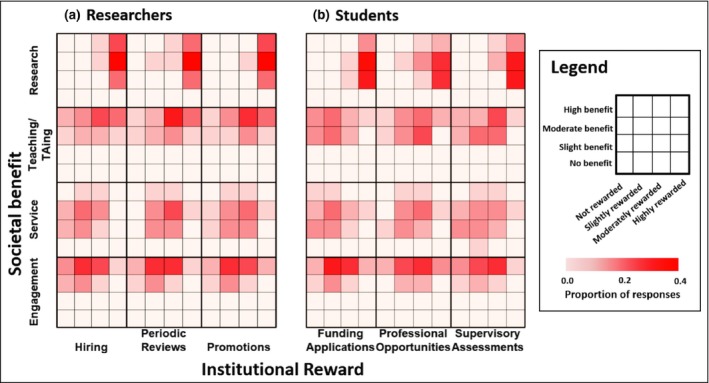
How (a) established researchers and (b) students perceive the societal benefits of four types of researcher activity (research, teaching, service, and engagement) and the degree to which their institutions reward each activity when carrying out processes related to hiring decisions, periodic reviews, and promotions (for established researchers) and processes related to funding applications, professional opportunities, and supervisory assessments (for students). Shades of red indicate the proportion of responses (along both dimensions of societal benefit and institutional reward) in each cell of the grid; darker reds denote higher proportion of responses. Because respondents rarely indicated that the societal benefits or institutional rewards of research activities were “unclear” (<4% of all responses), we do not show these responses in this figure.

Survey responses indicated that, with respect to each of the four categories that institutions generally measure and value (research, teaching, service, and engagement), there was a disparity between their perceived societal benefit and their perceived institutional reward. These results were consistent across nations, genders, and research disciplines, as well as between established researchers and students. Both established researchers and students mostly perceived engagement to have high societal benefit (with a minority perceiving research to have moderate societal benefit; Figure 2; WebTable 2); however, the apparent institutional reward varied from “not rewarded” to “highly rewarded”, with the most frequent response across career stages being “slightly rewarded” (Figure 2; WebTable 1). Research, on the other hand, was seen to be highly rewarded across multiple evaluation processes by a strong majority of established researchers and students, with few indicating research is moderately or slightly rewarded (Figure 2; WebTable 1). Established researchers reported greater perceived societal benefits of research than did students, although both groups viewed research as having less societal benefit than teaching and engagement (Figure 2; WebTable 2). The prevalence of the perception that research is highly rewarded is unsurprising given how many established metrics are used to judge research (eg publication counts, impact factors, *h*‐indices), which contribute to securing grants and increasing an institution's renown. Perhaps more unexpected is that research was not uniformly perceived to contribute high societal benefit (the most common responses were slight to moderate societal benefit). These results suggest some support for arguments that research without engagement leaves important scientific insights disconnected from real‐world impacts (Bowen and Graham 2013), or that there is a low likelihood that any individual research finding will benefit society (Nielsen 2001). Notably, on average, engagement (as perceived by both established researchers and students) was thought to benefit society to a similar degree as teaching (as perceived by established researchers only) but to a greater degree than research and service (as perceived by both established researchers and students) (Figure 2; WebTable 2).

Despite the perceived lack of institutional rewards for engagement, we found that 81% of the 315 research and scientific organizations represented in our sample included engagement, social service, or public dissemination within their mission statements, stated values, and/or organizational strategies (WebPanel 2). This suggests that mission statements do not alter the perception of whether institutions reward researchers’ engagement activities.

### Engagement: hardly any is more than enough

**Figure 3:**
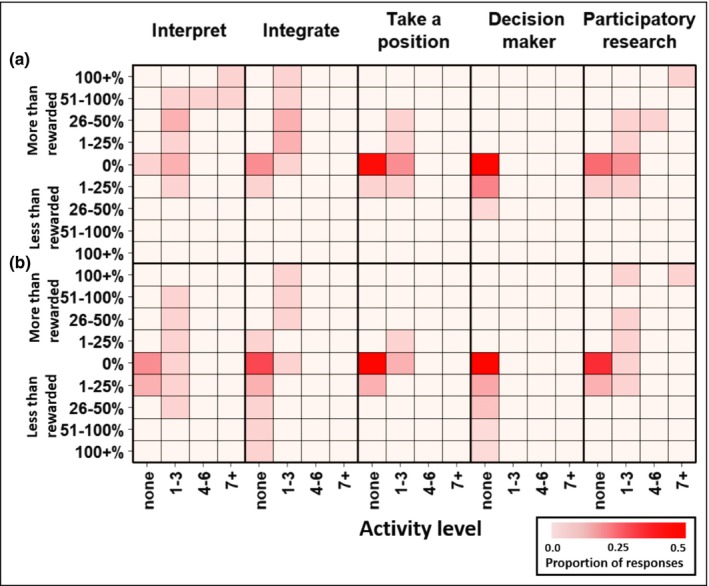
The degree to which (a) established researchers and (b) students think they are doing more or less engagement than is rewarded by their institutions. Darker reds denote higher proportion of responses. The x‐axis scores correspond with the number of engagement activities that respondents reported participating in during the past year, and the y‐axis scores correspond to either the amount of extra engagement (as percentages of current activity levels) that institutions would reward for (reported only by those who felt they were currently doing “less than rewarded”) or the amount of extra engagement (as percentages of the level that institutions reward for) that participants engage in above the maximum amount that their institutions reward for (reported only by those who felt they were currently doing “more than rewarded”). Some respondents who engaged in zero engagement activities commented on their survey that their institutions would reward for some engagement and so responded with positive “less than rewarded” values even though these should be technically “0%” (any proportion of 0 is 0%).

Across multiple categories of engagement, many established researchers and students indicated that they are doing more than their institutions will reward them for – this was true even for those who reported participating in just one to three engagement activities per year (Figure 3). Moreover, most individuals who reported zero engagement indicated that their institutions would not reward any level of engagement. For example, 52% of survey participants who indicated that they do not actively take a position on policy or act as a decision maker also reported that their institution would not reward them for doing so.

We found low levels of reported participation across multiple categories of engagement activity (Figure 3), which could be attributed to the paucity of institutional rewards for engagement. The relatively small number of researchers who reported taking a stand on policy positions or acting as a decision maker may also reflect the (contested) opinion that these activities can compromise academic rigor or integrity (Nielsen 2001; Kotcher *et al*. 2017), an opinion perhaps reinforced by the lack of rewards for these activities. Scientists worried about loss of credibility may also be hesitant to engage because engagement activities are often lumped together by institutional reporting systems as forms of “advocacy” even though some do not involve advocating for or against particular policies or approaches (Singh *et al*. 2014; Kotcher *et al*. 2017). Among the respondents who had actively taken a position or acted as a decision maker, 35% indicated that institutions should place more weight in these activities when evaluating individuals for career advancement, 31% suggested that institutions should employ additional metrics, 16% favored institutions placing higher expectations on these activities, and only 12% suggested that they should not be rewarded for these activities (note: those taking the survey were allowed to select more than one response).

### Many dimensions of engagement excellence are not assessed

**Figure 4:**
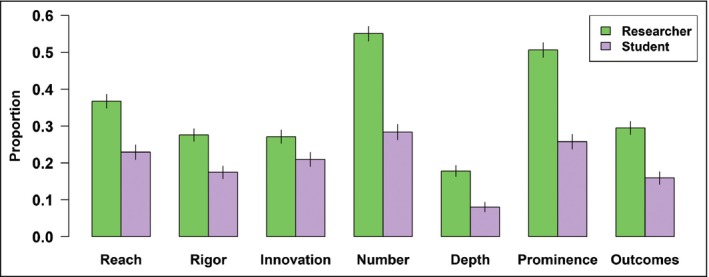
Perceptions of whether institutional evaluation captured each of our seven dimensions of engagement in various processes relevant to career advancement (eg 0.37 in the left‐most bar indicates that 37% of researcher respondents indicated that evaluations captured the *reach* of engagements, and 63% indicated they did not). Error bars represent standard errors.

Currently, most institutions have only unstructured ways to assess engagement, where it is assessed at all. Among respondents whose institutions assess engagement, 56% indicated that their institutions request qualitative, free‐written descriptions of the engagement activity. Although these free‐form evaluations are not in and of themselves problematic, researchers perceive that institutions evaluate these narratives using only a limited number of the seven dimensions of engagement we identify in this study (Table 1; Figure 4). Respondents indicated that their institutions predominantly assess engagement by the number of engagement activities undertaken, and how prominent the activities are, while rarely considering other dimensions (eg op‐eds in major newspapers are more prominent than posts on a seldom‐visited blog; Table 1). Relatively few respondents (30% of established researchers and 16% of students) reported that their institutions consider the actual outcomes of engagement activities. We defined “outcomes” of engagement as the things that change because of engagement; for example, have policy makers designed policy based on a policy brief they received?

Our results indicated that the dimensions of engagement addressed by current evaluations do not align with researcher motivations (ie the reasons why they chose to engage in the first place). For example, “prominence” (perceived esteem) was one of the most frequently evaluated dimensions of engagement but “status” (standing relative to other researchers) was generally cited as a weak motivator for engagement. In contrast, many researchers and students are motivated to educate or excite the public, to fulfill a sense of social responsibility, and to affect the wider world (Singh *et al*. 2014; Figure 4). In short, many established researchers and students engage (or wish to engage) specifically for the *outcomes* of engagement, while institutions often overlook these outcomes in their evaluations.

### Diverse and mainly selfless motivations drive engagement

**Figure 5:**
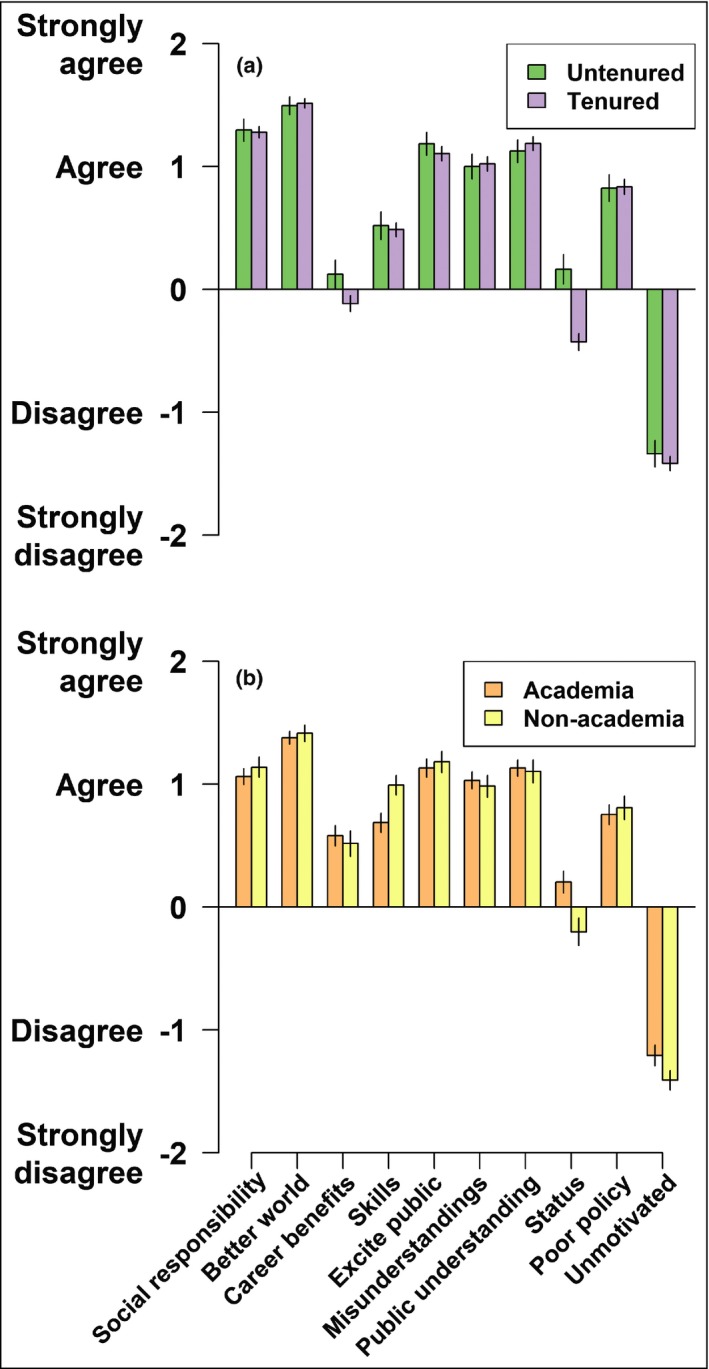
Reported agreement to statements about motivations to engage for (a) established researchers (from academic institutions) who were untenured (green/left bars) and tenured (lavender/right bars) and (b) students who intended to pursue careers in academia (orange/left bars) and outside of academia (yellow/right bars). Bars and error bars represent mean ± standard errors for each group's statements on motivations. Participants were asked to indicate their agreement with a series of statements (from left to right): I engage to fulfill a sense of social responsibility, to contribute to a better world, because it can lead to career benefits, to help develop transferable skills in communication, to excite the public about science and/or the humanities, to confront misunderstandings about science and/or the humanities, to build public understanding and/or trust in science, to raise my status as a research personality, to combat poor or ideological policy making, and I am not motivated to engage. We converted ordinal responses from the survey to numerical data for this figure to visualize the results according to the following scheme: for agreement (“strongly disagree” = –2, “disagree” = –1, “neutral” = 0, “agree” = 1, “strongly agree” = 2). For a discussion on treating ordinal data as interval/continuous data, please see WebPanel 2.

Researchers reported diverse motivations for engagement, most of which were “other‐oriented” (eg engaging to foster a better world, to fulfill a sense of social responsibility, to excite the public and build greater scientific understanding, to improve policy making; Figure 5). In contrast, very few researchers indicated that they are not motivated to engage at all. Across nations, career stages, and disciplines, “individual‐oriented” motivations (eg raising status as a research personality, developing communication skills, gaining career benefits) were the least important motivations stated by respondents (Figure 5). The prevalence of other‐oriented motivations found in this study agrees with the results of psychological research, which shows that acting on other‐oriented motivations provides individual benefits, such as fostering a sense of purpose and satisfying psychological and social needs (Crocker *et al*. 2017). Among respondents, only untenured researchers (ie those working toward promotion) and students intending to pursue careers in academia appeared to display an interest in engagement for personal gain (and these drivers remained less important than other‐oriented ones; Figure 5).

### Incentives and other‐oriented motives predict engagement activity

For both established researchers and students, perceived institutional rewards and stated motivations significantly predicted recent engagement activity. For established researchers (but not students), perceived institutional rewards for integrating research into policy, acting as a decision maker, and collaborating with communities on research projects were correlated with participation in those engagement activities (WebFigure 1). Perceived institutional rewards may not have predicted engagement by students because there was little variation in student responses regarding their participation in such activities, especially for acting as a decision maker (very few students indicated that they had acted in such a way). Few other‐oriented motivations positively predicted engagement behavior (one out of six other‐oriented motivations predicted activity across five engagement types for both established researchers and students). The motivation to combat poor or ideological policy making correlated positively with actively taking a policy position (for both established researchers and students; WebFigure 1). Only one out of three self‐oriented motivations was found to positively predict engagement activity, and only for established researchers. Those established researchers who were more motivated by the prospect of developing communication skills were more likely to conduct participatory research with communities (defined as directly involving communities or stakeholders in research design, execution, and/or knowledge dissemination; WebFigure 1). In contrast, for students, one out of three self‐oriented motivations negatively correlated with engagement activity. Those students indicating stronger motivations for career benefits were less likely to actively take a position (WebFigure 1). Similarly, agreeing with the statement “I am not motivated to engage” was negatively correlated with interpreting research for policy (for established researchers). Interestingly, self‐oriented motivations and perceived institutional reward did not positively correlate with activity for students, indicating that promoting engagement through career benefits, status, and so on may not be enough to promote engagement in students.

### Future directions

Addressing the discrepancies between societal benefits and institutional rewards as motivators is not straightforward. By incentivizing engagement, developing new metrics, or applying existing metrics more broadly, institutions may well encourage researchers to engage (Lane 2010). However, we recommend that three considerations be kept firmly in mind. First, evaluation processes that create self‐oriented motivations to promote or prevent activities may have unintended consequences (Bowles 2008). For example, introducing incentives and penalties may “crowd out” existing other‐oriented motivations (Gneezy and Rustichini 2000), potentially undermining or distorting the desired behavior. In a classic example of unintended consequences of introducing a self‐oriented penalty, when parents who did not pick up their children from daycare on time were made to pay a fine, the number of late‐arriving parents actually *increased* greatly, as parents could then simply pay for extra supervised time instead of striving to meet ethical obligations and avoid inconveniencing teachers (an other‐oriented motivation; Gneezy and Rustichini 2000). However, if correctly designed, incentives may actually leverage and augment existing motivations (Rode *et al*. 2015) and reinforce the prevailing notion that certain activities are socially beneficial. Incentives that are likely to leverage existing motivations are generally non‐monetary, and often involve public recognition, institutional metrics, and other signals that engagement is a socially desirable behavior. Stewardship awards for community‐based conservation activities are one example of an incentive that reinforces conservation motivations through appreciation (Chan *et al*. 2017).

Second, institutions would do well to mind Goodhart's Law: metrics adopted to assign rewards can quickly generate perverse outcomes as individuals seek to fulfill metrics that have become decoupled from their underlying intent (Elton 2004). Obsession with measurement can have numerous negative effects, including changing norms of accountability (Shore *et al*. 2015), and virtually all metrics are subject to such distortion when adopted as targets. One potential solution is to design adaptive processes that do not encourage fulfilling metrics as goals themselves.

Third, institutions should think beyond incentives. Behavior is more often a function of social structures and constraints than of incentives (Shove 2010). Removing constraints might take the form of department‐ or institution‐wide training, support and technical assistance for researchers to engage, and relief from other responsibilities (eg administrative tasks) to take on demanding engagement roles (eg in science–policy processes). Institutions can also promote ways to produce, share, and use policy‐relevant knowledge (Muñoz‐Erickson 2014) as a means to help scientists engage in policy and align research with societal values when scientists fear credibility loss as a result of anything associated with “advocacy”. With respect to affecting behavior, measurement programs may do so more through their articulation of values than by the programs’ tangible rewards or punishments (Vatn 2009, 2010).

Regardless of one's favored solutions, the discrepancies we highlight here put the onus squarely on institutions who are serious about societal benefit to reconsider their evaluation and reward structures regarding engagement (Carpini *et al*. 2004). Among those employed in research positions, we note widespread agreement that societal benefit is found not only (or even primarily) in research per se, but most strongly in teaching and engagement. Given that there is some evidence of a trade‐off between engagement activity and research output (Jin *et al*. 2016), rewarding engagement in addition to research can avoid putting the career advancement of researchers who engage at a disadvantage. In short, research institutions espousing public benefits would do well to acknowledge the importance of engagement and teaching, and to reward these activities commensurate with their importance to institutional missions.

Strong institutional support for engagement may be especially important to convey to students and other emerging researchers, who are often excited to engage but who face the apparent reality that only research is strongly valued by their institutions. Our results indicate that, despite this ostensible institutional value, students express uncertainty about the value of research to society. Perhaps students would be better served by institutional assessments that are reformulated to reflect not only the increasingly diverse research‐based professions outside of academia (Cyranoski *et al*. 2011) but also the strong motivations to engage. Doing so might help align institutions’ engagement processes with their mission statements, and the motivations of the next generation of researchers (Jin *et al*. 2016).

The need for engagement has never been more critical (Taylor 2007; Baron 2010, 2016; Richmond 2016). Nevertheless, research institutions’ current practices make such activities difficult, effectively imposing strong disincentives to spend time on tasks that are effectively uncompensated relative to research, which is consistently rewarded. However, rewarding research is not enough; now is the time to ensure that science *engagement* is appropriately enabled, evaluated, and rewarded as well.

## Supporting information

WebFigure 1

WebTable 1

WebTable 2

WebTable 3

WebTable 4

WebTable 5

WebPanel 1

WebPanel 2
