# Supplementary material for: Researcher engagement in policy deemed societally beneficial yet unrewarded
Source: Front Ecol Environ. 2019 Jul 30;17(7):375–82. doi: 10.1002/fee.2084 (PMC6910643; doi:10.1002/fee.2084)
Supplement: Supplementary file 1 — WebFigure 1 [file FEE-17-375-s001.pdf]

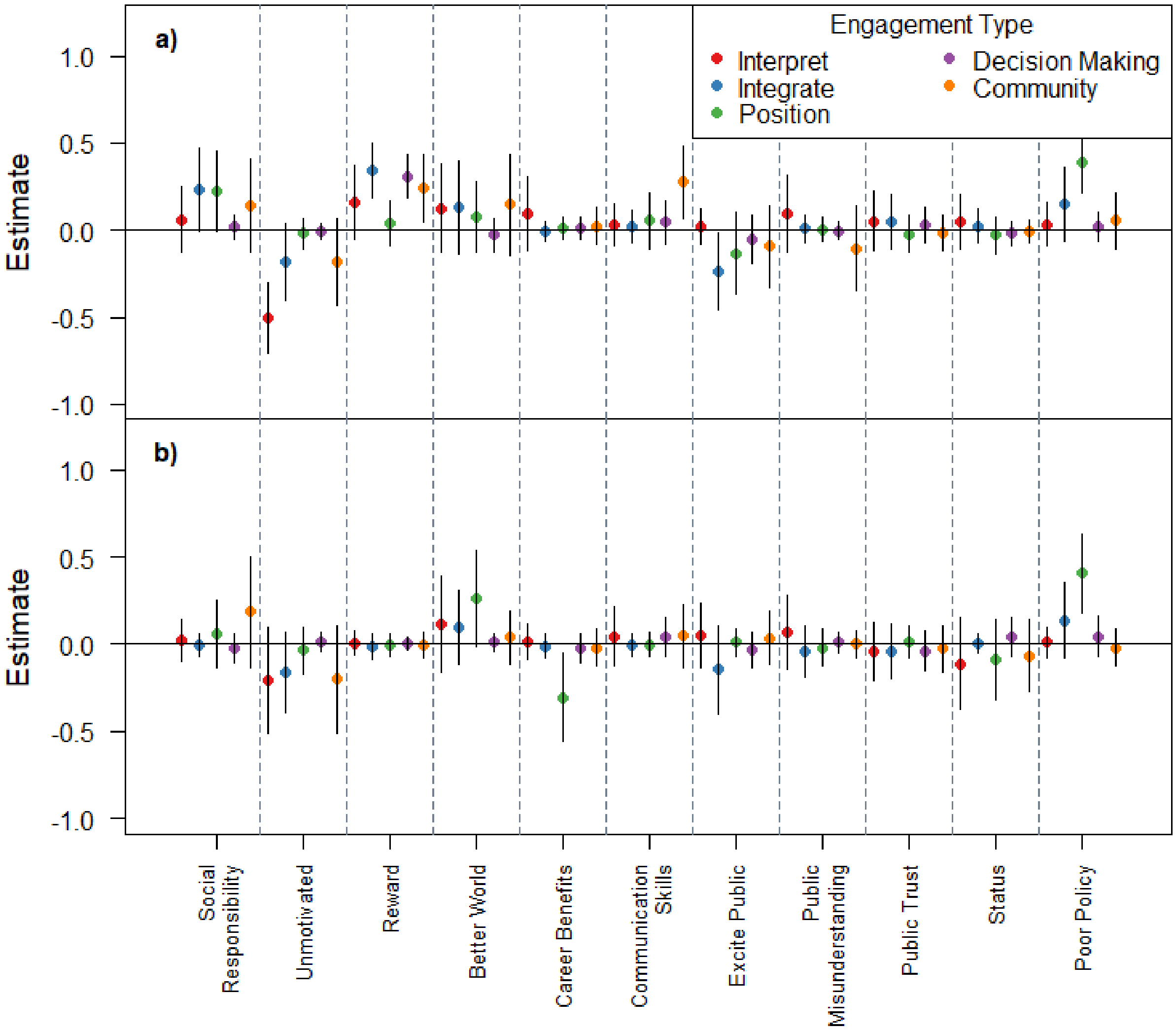


**WebFigure 1.** Regression coefficients for how nine motivations (in addition to perceptions of institutional reward, labeled as "reward") and being unmotivated explain variation in each type of engagement activity (interpret, integrate, taking a position, acting as a decision maker, and participatory research with communities). Points represent model-averaged standardized coefficient scores for each type of engagement shown in the key, and bars are 95% confidence intervals. Panels represent (a) established researchers and (b) students. Significant variables are those whose confidence intervals do not cross zero.
