# Supplementary material for: Researcher engagement in policy deemed societally beneficial yet unrewarded
Source: Front Ecol Environ. 2019 Jul 30;17(7):375–82. doi: 10.1002/fee.2084 (PMC6910643; doi:10.1002/fee.2084)
Supplement: Supplementary file 2 — WebTable 1 [file FEE-17-375-s002.pdf]

WebTable 1. Pair-wise comparison results of Tukey's tests (following ANOVA) of the perceived institutional reward for different activities for established researchers and students across different evaluation processes. We use a *P* value of 0.05 to determine statistical significance in Tukey tests. The same letters within a column indicate pairs that are statistically similar to each other ($P > 0.05$). Statistics for the ANOVA test are reported below this table.

Category	*Participant*	*Stage*	*Pairwise comparisons from ANOVA and Tukey's tests*																							
Research	Established researcher	Hiring	A																							
Research	Established researcher	Periodic reviews	A	B																						
Research	Established researcher	Promotion	A	B	C																					
Research	Student	Funding	A	B	C	D																				
Research	Student	Professional opportunities		B	C	D	E																			
Research	Student	Supervisory assessments	A	B	C	D	E	F																		
Teaching	Established researcher	Hiring							G																	
Teaching	Established researcher	Periodic reviews								H																
Teaching	Established researcher	Promotion									I															
TAing	Student	Funding										J														
TAing	Student	Professional opportunities											K													
TAing	Student	Supervisory assessments												L												
Internal service	Established researcher	Hiring										J		L	M											
Internal service	Established researcher	Periodic reviews							G				K	L		N										
Internal service	Established researcher	Promotion							G				K	L		N	O									
Internal service	Student	Funding										J			M			P								
Internal service	Student	Professional opportunities											K	L		N	O		Q							
Internal service	Student	Supervisory assessments										J		L	M			P		R						
Engagement	Established researcher	Hiring										J		L	M			P		R	S					
Engagement	Established researcher	Periodic reviews											K	L		N	O		Q		S	T				
Engagement	Established researcher	Promotion											K	L		N	O		Q	R	S	T	U			
Engagement	Student	Funding											K	L		N	O		Q		S	T	U	V		
Engagement	Student	Professional opportunities							G	H	I		K	L		N	O		Q		S	T		V	W	
Engagement	Student	Supervisory assessments											K	L	M	N	O		Q	R	S	T	U	V	W	X

Notes: Individual letters within columns indicate perceived reward for the rows that are statistically similar to each other. Comparisons are pairwise, with specific rows compared to the uppermost row with a given letter within a given column; for example, on average students perceive research to be rewarded similarly for supervisory assessments as established researchers perceive research to be rewarded for hiring (both activities have the letter "A", where "A" indicates similarity in pairwise comparisons with establish research perceptions on reward for research in hiring). In contrast, students do not perceive research to be rewarded for professional opportunities similarly to how established researchers perceive research to be rewarded for hiring (student perceptions of reward for research for professional opportunities does not have an "A" indicating no similarity in pairwise comparison with established researcher perceptions of reward for research in hiring) . TA = teaching assistant.

ANOVA table

	**df**	**Sum of squares**	**Mean Square**	***F* test**	***P* value**
Categories	23	3420	148.72	194.1	<2e-16
Residuals	11466	8785	0.77		
