# Supplementary material for: Researcher engagement in policy deemed societally beneficial yet unrewarded
Source: Front Ecol Environ. 2019 Jul 30;17(7):375–82. doi: 10.1002/fee.2084 (PMC6910643; doi:10.1002/fee.2084)
Supplement: Supplementary file 3 — WebTable 2 [file FEE-17-375-s003.pdf]

GG Singh *et al*. – Supporting Information

WebTable 2. Pair-wise comparison results of Tukey's test results (following ANOVA) of the perceived societal benefit of different activities for established researchers and students. We use a P value of 0.05 to determine statistical significance in Tukey tests. The same letters within a column indicate pairs that are statistically similar to each other ($P > 0.05$).

Category	*Participant*	*Tukey's test results*							
Research	Established researcher	A							
Research	Student		B						
Teaching	Established researcher			C					
TAing	Student				D				
Internal service	Established researcher					E			
Internal service	Student					E	F		
Engagement	Established researcher			C				G	
Engagement	Student			C				G	H

Notes: Individual letters within columns indicate perceived societal benefit of rows that are statistically similar to one another. Comparisons are pairwise, with specific rows compared to the uppermost row with a given letter within a given column; for example, on average both established researchers and students perceive engagement to have as much societal benefit as established researchers perceive for teaching (established researchers and student perceptions on engagement have the letter "C" meaning that each is similar to how established researchers perceive the societal benefit of teaching). TA = teaching assistant.

ANOVA table

	**df**	**Sum of squares**	**Mean square**	***F* test**	***P* value**
Categories	7	664.1	94.87	182.5	<2e-16
Residuals	4080	2120.3	0.52		
