# Supplementary material for: Researcher engagement in policy deemed societally beneficial yet unrewarded
Source: Front Ecol Environ. 2019 Jul 30;17(7):375–82. doi: 10.1002/fee.2084 (PMC6910643; doi:10.1002/fee.2084)
Supplement: Supplementary file 4 — WebTable 3 [file FEE-17-375-s004.pdf]

WebTable 3. Demographics of survey participants

	Established researchers (*n = 634)*	*Students* (*n = 458)*
**Institution**		
Academic	69.87	38.43
Agency (federal/provincial/state)	7.73	7.64
Non-governmental organization	4.89	6.33
Private company	0.47	3.49
Research council	0.47	0.00
Parastatal	0.16	0.00
Freelance	0.16	0.66
Law	0.00	0.22
Political	0.00	0.22
Hospital	0.00	0.22
Education	0.00	0.44
Museum	0.00	0.22
Unsure	0.00	4.15
No response	16.25	37.99
**Discipline**		
Applied science/engineering	4.73	3.06
Health sciences	5.05	4.15
Humanities	1.10	0.44
Interdisciplinary	9.94	10.70
Natural sciences	51.10	39.30
Physical sciences	3.79	0.22
Social sciences	6.94	5.46
Mathematics	0.16	0.22
Linguistics	0.16	0.00
Arts	0.16	0.00
Industrial design	0.00	0.22
No response	16.88	36.24
**Gender**		
Female	37.70	40.39
Male	44.64	23.14
Other	0.32	0.22
No response	17.35	36.24
**Country/territory**		
Australia	3.15	3.71
Austria	0.32	0.00
Bangladesh	0.16	0.00
Belgium	1.74	0.22
Benin	0.32	0.00
Bolivia	0.00	0.22
Brazil	7.10	3.93
Canada	16.09	25.55
Chile	0.79	0.44

China	0.32	0.44
Colombia	0.00	0.22
Croatia	0.16	0.00
Denmark	0.32	0.44
Ecuador	0.16	0.00
Egypt	0.16	0.00
Ethiopia	0.16	0.00
Finland	0.16	0.44
France	0.16	0.00
Germany	0.95	0.87
Ghana	0.00	0.22
Greece	0.16	0.22
Iceland	0.16	0.00
India	0.63	0.44
Ireland	0.16	0.44
Israel	0.63	0.66
Italy	0.95	0.87
Japan	2.37	1.09
Jordan	0.32	0.00
Kenya	0.16	0.00
Kyrgyzstan	0.00	0.22
Malaysia	0.47	0.00
Mauritius	0.16	0.00
Mexico	0.63	0.22
Morocco	0.00	0.22
Netherlands	0.32	0.87
New Zealand	0.95	0.22
Nigeria	0.32	0.00
Pakistan	0.16	0.00
Peru	0.16	0.00
Philippines	0.32	0.00
Puerto Rico	0.16	0.00
Serbia	0.47	1.09
Slovakia	0.16	0.00
South Africa	3.94	6.11
Spain	0.95	1.75
Sweden	0.32	1.97
Switzerland	0.95	1.09
Taiwan	0.16	0.00
Thailand	0.32	0.22
Turkey	1.89	4.80
United Arab Emirates	0.16	0.00
UK	4.42	4.15
Uruguay	0.32	0.66
US (excluding Puerto Rico)	36.12	24.02
Venezuela	0.00	0.22
Not given	8.52	11.79

Notes: All values shown are percentages (%).
