# Supplementary material for: Researcher engagement in policy deemed societally beneficial yet unrewarded
Source: Front Ecol Environ. 2019 Jul 30;17(7):375–82. doi: 10.1002/fee.2084 (PMC6910643; doi:10.1002/fee.2084)
Supplement: Supplementary file 5 — WebTable 4 [file FEE-17-375-s005.pdf]

WebTable 4. The top 20 candidate models included in each average model for established researchers across five categories of engagement, with their associated corrected Akaike's information criterion (AICc) scores (as well as delta AICc from the top ranked model) and model weights

**Interpret science for policy makers and the public**					
Candidate model rank	Variables in candidate model	AICc	Delta AICc	Akaike weights	Cumulative weight
1	Better World + Career Benefits + Public Misunderstandings + Unmotivated + Reward	1326.320	0.000	0.016	0.016
2	Social Responsibility + Career Benefits + Public Misunderstandings + Unmotivated + Reward	1326.745	0.425	0.013	0.028
3	Social Responsibility + Better World + Career Benefits + Public Misunderstandings + Unmotivated + Reward	1327.128	0.808	0.010	0.038
4	Better World + Career Benefits + Public Misunderstandings + Poor Policy + Unmotivated + Reward	1327.743	1.423	0.008	0.046
5	Social Responsibility + Career Benefits + Public Trust + Unmotivated + Reward	1327.749	1.430	0.008	0.054
6	Social Responsibility + Career Benefits + Public Misunderstandings + Poor Policy + Unmotivated + Reward	1327.785	1.465	0.007	0.061
7	Better World + Career Benefits + Communication Skills + Public Misunderstandings + Unmotivated + Reward	1327.850	1.530	0.007	0.068
8	Better World + Career Benefits + Public Misunderstandings + Status + Unmotivated + Reward	1327.946	1.627	0.007	0.075
9	Better World + Career Benefits + Public Misunderstandings + Public Trust + Unmotivated + Reward	1327.960	1.640	0.007	0.082
10	Better World + Public Misunderstandings + Status + Unmotivated + Reward	1328.000	1.680	0.007	0.089
11	Social Responsibility + Career Benefits + Public Misunderstandings + Public Trust + Unmotivated + Reward	1328.074	1.754	0.006	0.095
12	Better World + Career Benefits + Public Trust + Unmotivated + Reward	1328.164	1.844	0.006	0.101
13	Social Responsibility + Career Benefits + Public Trust + Poor Policy + Unmotivated + Reward	1328.199	1.879	0.006	0.107
14	Better World + Career Benefits + Public Misunderstandings + Unmotivated	1328.320	2.000	0.006	0.113
15	Social Responsibility + Career Benefits + Public Misunderstandings + Status + Unmotivated + Reward	1328.322	2.002	0.006	0.119
16	Social Responsibility + Career Benefits + Public Misunderstandings + Unmotivated	1328.341	2.021	0.006	0.124
17	Better World + Career Benefits + Excite Public + Public Misunderstandings + Unmotivated + Reward	1328.384	2.065	0.006	0.130
18	Better World + Public Misunderstandings + Unmotivated + Reward	1328.386	2.066	0.006	0.135
19	Social Responsibility + Career Benefits + Communication Skills + Public Misunderstandings + Unmotivated + Reward	1328.403	2.083	0.005	0.141
20	Social Responsibility + Better World + Career Benefits + Public Trust + Unmotivated + Reward	1328.573	2.253	0.005	0.146
2048	NULL	1390.717	64.398	0.000	

Integrate science into decision-making					
Candidate model rank	Variables in candidate model	AICc	Delta AICc	Akaike weights	Cumulative weight
1	Social Responsibility + Better World + Excite Public + Poor Policy + Unmotivated + Reward	1233.803	0.000	0.044	0.044
2	Social Responsibility + Excite Public + Poor Policy + Unmotivated + Reward	1233.869	0.066	0.043	0.087
3	Social Responsibility + Excite Public + Public Trust + Poor Policy + Unmotivated + Reward	1234.775	0.972	0.027	0.114
4	Social Responsibility + Better World + Excite Public + Status + Poor Policy + Unmotivated + Reward	1234.859	1.056	0.026	0.140
5	Social Responsibility + Excite Public + Status + Poor Policy + Unmotivated + Reward	1234.906	1.102	0.025	0.165
6	Social Responsibility + Better World + Excite Public + Public Trust + Poor Policy + Unmotivated + Reward	1235.009	1.206	0.024	0.189
7	Social Responsibility + Better World + Communication Skills + Excite Public + Poor Policy + Unmotivated + Reward	1235.094	1.291	0.023	0.212
8	Social Responsibility + Communication Skills + Excite Public + Poor Policy + Unmotivated + Reward	1235.200	1.397	0.022	0.234
9	Social Responsibility + Better World + Excite Public + Public Misunderstandings + Poor Policy + Unmotivated + Reward	1235.688	1.884	0.017	0.251
10	Social Responsibility + Excite Public + Public Trust + Status + Poor Policy + Unmotivated + Reward	1235.727	1.923	0.017	0.268
11	Social Responsibility + Career Benefits + Excite Public + Poor Policy + Unmotivated + Reward	1235.782	1.978	0.016	0.285
12	Social Responsibility + Better World + Career Benefits + Excite Public + Poor Policy + Unmotivated + Reward	1235.794	1.991	0.016	0.301
13	Social Responsibility + Excite Public + Public Misunderstandings + Poor Policy + Unmotivated + Reward	1235.817	2.014	0.016	0.317
14	Social Responsibility + Better World + Excite Public + Public Trust + Status + Poor Policy + Unmotivated + Reward	1235.993	2.189	0.015	0.332
15	Social Responsibility + Communication Skills + Excite Public + Public Trust + Poor Policy + Unmotivated + Reward	1236.278	2.475	0.013	0.345
16	Social Responsibility + Better World + Communication Skills + Excite Public + Status + Poor Policy + Unmotivated + Reward	1236.367	2.563	0.012	0.357
17	Social Responsibility + Communication Skills + Excite Public + Status + Poor Policy + Unmotivated + Reward	1236.447	2.644	0.012	0.369
18	Social Responsibility + Better World + Communication Skills + Excite Public + Public Trust + Poor Policy + Unmotivated + Reward	1236.459	2.655	0.012	0.380
19	Social Responsibility + Career Benefits + Excite Public + Public Trust + Poor Policy + Unmotivated + Reward	1236.713	2.910	0.010	0.391
20	Social Responsibility + Better World + Excite Public + Public Misunderstandings + Status + Poor Policy + Unmotivated + Reward	1236.756	2.953	0.010	0.401
2010	NULL	1286.899	53.095	0.000	

**Actively take a position**					
Candidate model rank	Variables in candidate model	AICc	Delta AICc	Akaike weights	Cumulative weight
1	Social Responsibility + Communication Skills + Excite Public + Poor Policy	1262.375	0.000	0.024	0.024
2	Social Responsibility + Excite Public + Poor Policy	1262.604	0.230	0.022	0.046
3	Social Responsibility + Better World + Communication Skills + Excite Public + Poor Policy	1262.940	0.565	0.018	0.064
4	Social Responsibility + Better World + Excite Public + Poor Policy	1263.167	0.793	0.016	0.081
5	Social Responsibility + Communication Skills + Excite Public + Status + Poor Policy	1263.451	1.076	0.014	0.095
6	Social Responsibility + Communication Skills + Excite Public + Poor Policy + Reward	1263.921	1.546	0.011	0.106
7	Social Responsibility + Excite Public + Poor Policy + Reward	1263.953	1.578	0.011	0.117
8	Social Responsibility + Communication Skills + Excite Public + Poor Policy + Unmotivated	1263.997	1.622	0.011	0.128
9	Social Responsibility + Better World + Communication Skills + Excite Public + Status + Poor Policy	1264.010	1.635	0.011	0.139
10	Social Responsibility + Excite Public + Status + Poor Policy	1264.065	1.691	0.010	0.149
11	Social Responsibility + Excite Public + Poor Policy + Unmotivated	1264.098	1.723	0.010	0.160
12	Social Responsibility + Communication Skills + Excite Public + Public Misunderstandings + Poor Policy	1264.345	1.971	0.009	0.169
13	Social Responsibility + Communication Skills + Excite Public + Public Trust + Poor Policy	1264.384	2.009	0.009	0.178
14	Social Responsibility + Career Benefits + Communication Skills + Excite Public + Poor Policy	1264.411	2.036	0.009	0.186
15	Social Responsibility + Career Benefits + Excite Public + Poor Policy	1264.411	2.037	0.009	0.195
16	Social Responsibility + Better World + Communication Skills + Excite Public + Poor Policy + Reward	1264.477	2.103	0.009	0.204
17	Social Responsibility + Excite Public + Public Misunderstandings + Poor Policy	1264.484	2.109	0.008	0.212
18	Social Responsibility + Better World + Excite Public + Poor Policy + Reward	1264.504	2.130	0.008	0.221
19	Social Responsibility + Better World + Excite Public + Status + Poor Policy	1264.626	2.251	0.008	0.229
20	Social Responsibility + Excite Public + Public Trust + Poor Policy	1264.651	2.276	0.008	0.236
1954	NULL	1302.530	40.155	0.000	

Act as a decision maker with regard to policy					
Candidate model rank	Variables in candidate model	AICc	Delta AICc	Akaike weights	Cumulative weight
1	Communication Skills + Excite Public + Reward	963.989	0.000	0.016	0.016
2	Communication Skills + Reward	964.361	0.371	0.013	0.029
3	Reward	964.648	0.659	0.011	0.040
4	Communication Skills + Excite Public + Public Trust + Reward	965.054	1.064	0.009	0.049
5	Communication Skills + Excite Public + Poor Policy + Reward	965.128	1.139	0.009	0.058
6	Better World + Communication Skills + Reward	965.569	1.580	0.007	0.065
7	Poor Policy + Reward	965.679	1.690	0.007	0.072
8	Career Benefits + Communication Skills + Excite Public + Reward	965.737	1.748	0.007	0.079
9	Excite Public + Reward	965.739	1.750	0.007	0.085
10	Better World + Communication Skills + Excite Public + Reward	965.747	1.757	0.007	0.092
11	Communication Skills + Excite Public + Status + Reward	965.776	1.787	0.006	0.098
12	Communication Skills + Public Misunderstandings + Reward	965.785	1.795	0.006	0.105
13	Social Responsibility + Communication Skills + Excite Public + Reward	965.823	1.833	0.006	0.111
14	Communication Skills + Poor Policy + Reward	965.844	1.855	0.006	0.117
15	Communication Skills + Status + Reward	965.872	1.883	0.006	0.123
16	Communication Skills + Excite Public + Unmotivated + Reward	965.923	1.934	0.006	0.129
17	Communication Skills + Excite Public + Public Misunderstandings + Reward	966.039	2.049	0.006	0.135
18	Career Benefits + Reward	966.131	2.142	0.005	0.140
19	Excite Public + Public Trust + Reward	966.226	2.237	0.005	0.145
20	Career Benefits + Communication Skills + Reward	966.278	2.288	0.005	0.150
1056	NULL	987.122	23.132	0.000	

Participatory research involving communities or stakeholders					
Candidate model rank	Variables in candidate model	AICc	Delta AICc	Akaike weights	Cumulative weight
1	Social Responsibility + Better World + Communication Skills + Excite Public + Unmotivated + Reward	1349.964	0.000	0.026	0.026
2	Social Responsibility + Communication Skills + Excite Public + Unmotivated + Reward	1350.240	0.276	0.023	0.049
3	Better World + Communication Skills + Excite Public + Unmotivated + Reward	1350.245	0.281	0.023	0.072
4	Social Responsibility + Better World + Communication Skills + Excite Public + Public Misunderstandings + Unmotivated + Reward	1350.942	0.978	0.016	0.088
5	Social Responsibility + Communication Skills + Excite Public + Public Misunderstandings + Unmotivated + Reward	1351.208	1.244	0.014	0.102
6	Social Responsibility + Communication Skills + Public Misunderstandings + Unmotivated + Reward	1351.322	1.358	0.013	0.116
7	Social Responsibility + Communication Skills + Public Misunderstandings + Poor Policy + Unmotivated + Reward	1351.341	1.377	0.013	0.129
8	Social Responsibility + Communication Skills + Excite Public + Poor Policy + Unmotivated + Reward	1351.415	1.451	0.013	0.142
9	Better World + Communication Skills + Excite Public + Public Misunderstandings + Unmotivated + Reward	1351.537	1.573	0.012	0.154
10	Social Responsibility + Communication Skills + Excite Public + Public Misunderstandings + Poor Policy + Unmotivated + Reward	1351.582	1.618	0.012	0.166
11	Social Responsibility + Better World + Communication Skills + Excite Public + Poor Policy + Unmotivated + Reward	1351.597	1.633	0.012	0.177
12	Social Responsibility + Better World + Communication Skills + Public Misunderstandings + Unmotivated + Reward	1351.607	1.643	0.012	0.189
13	Social Responsibility + Better World + Career Benefits + Communication Skills + Excite Public + Unmotivated + Reward	1351.733	1.769	0.011	0.200
14	Better World + Communication Skills + Excite Public + Poor Policy + Unmotivated + Reward	1351.790	1.826	0.011	0.210
15	Social Responsibility + Better World + Communication Skills + Excite Public + Public Trust + Unmotivated + Reward	1351.827	1.863	0.010	0.221
16	Social Responsibility + Career Benefits + Communication Skills + Excite Public + Unmotivated + Reward	1351.849	1.885	0.010	0.231
17	Social Responsibility + Better World + Communication Skills + Excite Public + Reward	1351.947	1.983	0.010	0.241
18	Social Responsibility + Better World + Communication Skills + Excite Public + Public Misunderstandings + Poor Policy + Unmotivated + Reward	1351.985	2.021	0.010	0.250
19	Social Responsibility + Better World + Communication Skills + Excite Public + Status + Unmotivated + Reward	1352.031	2.067	0.009	0.260
20	Better World + Communication Skills + Excite Public + Public Trust + Unmotivated + Reward	1352.138	2.174	0.009	0.269
2011	NULL	1384.768	34.804	0.000	

Notes: For comparison, the null model associated with each engagement type is shown as well.
