# Supplementary material for: Researcher engagement in policy deemed societally beneficial yet unrewarded
Source: Front Ecol Environ. 2019 Jul 30;17(7):375–82. doi: 10.1002/fee.2084 (PMC6910643; doi:10.1002/fee.2084)
Supplement: Supplementary file 6 — WebTable 5 [file FEE-17-375-s006.pdf]

WebTable 5. The top 20 candidate models included in each average model for students across five categories of engagement, with their associated corrected Akaike's information criterion (AICc) scores, as well as delta AICc from the top ranked model and model weights

**Interpret science for policy makers and the public**					
Candidate model rank	Variables in candidate model	AICc	Delta AICc	Akaike weights	Cumulative weight
1	Better World + Unmotivated	659.835	0.000	0.016	0.016
2	Better World + Public Misunderstanding + Unmotivated	660.928	1.093	0.009	0.026
3	Better World + Public Misunderstanding + Public Trust + Unmotivated	661.042	1.206	0.009	0.035
4	Better World + Status + Unmotivated	661.062	1.226	0.009	0.043
5	Better World + Communication Skills + Unmotivated	661.420	1.585	0.007	0.051
6	Better World + Public Trust + Unmotivated	661.517	1.681	0.007	0.058
7	Better World + Unmotivated + Reward	661.671	1.835	0.007	0.064
8	Public Misunderstanding + Unmotivated	661.680	1.845	0.006	0.071
9	Better World + Excite Public + Unmotivated	661.793	1.957	0.006	0.077
10	Social Responsibility + Better World + Unmotivated	661.794	1.958	0.006	0.083
11	Better World + Career Benefits + Unmotivated	661.824	1.989	0.006	0.089
12	Unmotivated	661.848	2.013	0.006	0.095
13	Better World + Poor Policy + Unmotivated	661.853	2.017	0.006	0.101
14	Better World + Public Misunderstanding + Status + Unmotivated	661.892	2.056	0.006	0.107
15	Better World + Communication Skills + Status + Unmotivated	661.900	2.064	0.006	0.113
16	Better World + Public Misunderstanding + Public Trust + Status + Unmotivated	662.306	2.471	0.005	0.117
17	Social Responsibility + Unmotivated	662.395	2.559	0.005	0.122
18	Public Misunderstanding + Status + Unmotivated	662.429	2.594	0.004	0.126
19	Public Misunderstanding + Public Trust + Unmotivated	662.433	2.597	0.004	0.131
20	Better World + Communication Skills + Public Misunderstanding + Public Trust + Unmotivated	662.466	2.631	0.004	0.135
1420	NULL	670.315	10.480	0.000	

Integrate science into decision-making					
Candidate model rank	Variables in candidate model	AICc	Delta AICc	Akaike weights	Cumulative weight
1	Excite Public + Poor Policy + Unmotivated	506.169	0.000	0.025	0.025
2	Better World + Excite Public + Poor Policy + Unmotivated	506.610	0.440	0.020	0.046
3	Excite Public + Public Trust + Poor Policy + Unmotivated	507.399	1.230	0.014	0.059
4	Better World + Excite Public + Public Trust + Poor Policy + Unmotivated	507.437	1.267	0.013	0.073
5	Excite Public + Public Misunderstanding + Poor Policy + Unmotivated	507.541	1.371	0.013	0.085
6	Better World + Excite Public + Public Misunderstanding + Poor Policy + Unmotivated	507.608	1.439	0.012	0.098
7	Excite Public + Status + Poor Policy + Unmotivated	508.039	1.870	0.010	0.108
8	Excite Public + Poor Policy + Unmotivated + Reward	508.134	1.965	0.009	0.117
9	Career Benefits + Excite Public + Poor Policy + Unmotivated	508.136	1.967	0.009	0.126
10	Communication Skills + Excite Public + Poor Policy + Unmotivated	508.240	2.071	0.009	0.135
11	Social Responsibility + Excite Public + Poor Policy + Unmotivated	508.266	2.096	0.009	0.144
12	Social Responsibility + Better World + Excite Public + Poor Policy + Unmotivated	508.304	2.134	0.009	0.153
13	Public Trust + Poor Policy + Unmotivated	508.346	2.176	0.009	0.161
14	Better World + Excite Public + Status + Poor Policy + Unmotivated	508.359	2.190	0.008	0.170
15	Better World + Career Benefits + Excite Public + Poor Policy + Unmotivated	508.511	2.341	0.008	0.178
16	Better World + Excite Public + Poor Policy + Unmotivated + Reward	508.654	2.485	0.007	0.185
17	Better World + Public Trust + Poor Policy + Unmotivated	508.676	2.506	0.007	0.192
18	Better World + Communication Skills + Excite Public + Poor Policy + Unmotivated	508.726	2.557	0.007	0.199
19	Better World + Excite Public + Public Trust + Status + Poor Policy + Unmotivated	509.054	2.885	0.006	0.205
20	Better World + Excite Public + Public Misunderstanding + Public Trust + Poor Policy + Unmotivated	509.097	2.928	0.006	0.211
901	NULL	516.579	10.409	0.000	

**Actively take a position**					
Candidate model rank	Variables in candidate model	AICc	Delta AICc	Akaike weights	Cumulative weight
1	Better World + Career Benefits + Poor Policy	615.558	0.000	0.038	0.038
2	Better World + Career Benefits + Status + Poor Policy	615.987	0.430	0.031	0.069
3	Social Responsibility + Better World + Career Benefits + Poor Policy	616.204	0.647	0.028	0.096
4	Social Responsibility + Better World + Career Benefits + Status + Poor Policy	616.958	1.401	0.019	0.115
5	Better World + Career Benefits + Poor Policy + Unmotivated	617.214	1.656	0.017	0.132
6	Better World + Career Benefits + Public Trust + Poor Policy	617.420	1.862	0.015	0.147
7	Better World + Career Benefits + Public Misunderstanding + Poor Policy	617.460	1.902	0.015	0.161
8	Better World + Career Benefits + Excite Public + Poor Policy	617.545	1.987	0.014	0.175
9	Better World + Career Benefits + Poor Policy + Reward	617.593	2.036	0.014	0.189
10	Better World + Career Benefits + Communication Skills + Poor Policy	617.649	2.091	0.013	0.203
11	Better World + Career Benefits + Status + Poor Policy + Unmotivated	617.769	2.212	0.013	0.215
12	Better World + Career Benefits + Public Trust + Status + Poor Policy	617.804	2.247	0.012	0.228
13	Better World + Career Benefits + Public Misunderstanding + Status + Poor Policy	617.950	2.392	0.011	0.239
14	Better World + Career Benefits + Excite Public + Status + Poor Policy	617.977	2.420	0.011	0.250
15	Better World + Career Benefits + Status + Poor Policy + Reward	618.039	2.481	0.011	0.261
16	Social Responsibility + Better World + Career Benefits + Public Misunderstanding + Poor Policy	618.054	2.496	0.011	0.272
17	Better World + Career Benefits + Communication Skills + Status + Poor Policy	618.073	2.516	0.011	0.283
18	Social Responsibility + Better World + Career Benefits + Poor Policy + Unmotivated	618.126	2.569	0.011	0.294
19	Social Responsibility + Better World + Career Benefits + Poor Policy + Reward	618.223	2.665	0.010	0.304
20	Social Responsibility + Better World + Career Benefits + Communication Skills + Poor Policy	618.226	2.669	0.010	0.314
1914	NULL	646.252	30.695	0.000	

**Act as a decision maker with regard to policy**					
Candidate model rank	Variables in candidate model	AICc	Delta AICc	Akaike weights	Cumulative weight
1	Public Trust + Status + Poor Policy	291.639	0.000	0.006	0.006
2	Public Trust + Poor Policy	291.783	0.144	0.006	0.011
3	Public Trust + Status	291.957	0.318	0.005	0.017
4	Status	292.004	0.365	0.005	0.021
5	Career Benefits + Status	292.203	0.564	0.004	0.026
6	Excite Public + Status	292.250	0.611	0.004	0.030
7	Communication Skills + Public Trust + Poor Policy	292.382	0.743	0.004	0.034
8	Poor Policy	292.399	0.760	0.004	0.038
9	NULL	292.477	0.839	0.004	0.042
10	Excite Public + Poor Policy	292.578	0.939	0.004	0.046
11	Career Benefits + Public Trust + Status + Poor Policy	292.591	0.953	0.004	0.050
12	Excite Public + Status + Poor Policy	292.656	1.017	0.004	0.053
13	Status + Poor Policy	292.667	1.028	0.004	0.057
14	Social Responsibility + Poor Policy	292.690	1.052	0.004	0.060
15	Career Benefits + Public Trust + Status	292.766	1.128	0.003	0.064
16	Communication Skills + Excite Public + Poor Policy	292.804	1.165	0.003	0.067
17	Communication Skills + Excite Public	292.848	1.209	0.003	0.070
18	Career Benefits + Status + Poor Policy	292.871	1.233	0.003	0.073
19	Career Benefits + Excite Public + Status	292.969	1.330	0.003	0.077
20	Excite Public	292.997	1.358	0.003	0.080

**Participatory research involving communities or stakeholders**					
Candidate model rank	Variables in candidate model	AICc	Delta AICc	Akaike weights	Cumulative weight
1	Social Responsibility + Unmotivated	678.963	0.000	0.014	0.014
2	Social Responsibility + Status + Unmotivated	679.752	0.788	0.009	0.023
3	Social Responsibility + Excite Public + Unmotivated	680.424	1.460	0.007	0.030
4	Unmotivated	680.434	1.470	0.007	0.037
5	Social Responsibility + Career Benefits + Unmotivated	680.440	1.477	0.007	0.043
6	Social Responsibility + Poor Policy + Unmotivated	680.502	1.538	0.006	0.050
7	Social Responsibility + Public Trust + Unmotivated	680.534	1.571	0.006	0.056
8	Social Responsibility + Excite Public + Public Trust + Unmotivated	680.583	1.620	0.006	0.062
9	Social Responsibility + Communication Skills + Unmotivated	680.802	1.839	0.006	0.068
10	Social Responsibility + Communication Skills + Status + Unmotivated	680.852	1.889	0.005	0.073
11	Social Responsibility + Better World + Unmotivated	680.863	1.899	0.005	0.079
12	Social Responsibility + Unmotivated + Reward	680.879	1.915	0.005	0.084
13	Status + Unmotivated	680.881	1.918	0.005	0.089
14	Social Responsibility + Public Misunderstanding + Unmotivated	680.891	1.927	0.005	0.095
15	Communication Skills + Status + Unmotivated	680.958	1.995	0.005	0.100
16	Better World + Unmotivated	680.970	2.006	0.005	0.105
17	Social Responsibility + Excite Public + Status + Unmotivated	681.015	2.051	0.005	0.110
18	Excite Public + Unmotivated	681.264	2.301	0.004	0.114
19	Excite Public + Status + Unmotivated	681.445	2.482	0.004	0.118
20	Social Responsibility	681.455	2.492	0.004	0.122
771	NULL	686.350	7.386	0.000	

Notes: For comparison, the null model associated with each engagement type is shown as well.
