# Supplementary material for: Researcher engagement in policy deemed societally beneficial yet unrewarded
Source: Front Ecol Environ. 2019 Jul 30;17(7):375–82. doi: 10.1002/fee.2084 (PMC6910643; doi:10.1002/fee.2084)
Supplement: Supplementary file 7 — WebPanel 1 [file FEE-17-375-s007.pdf]

## WebPanel 1. The two surveys sent out to established researchers and students.

## Part 1: Survey sent to established researchers

### Scientists and engagement/outreach survey

There are relatively well-developed metrics for some research and teaching activities of scientists (scientists interpreted most broadly to include scholars of all kinds), but other activities have received relatively less attention. This study focuses on engagement or outreach – communicating or working with people outside of research institutions (eg journalists, industry, policy makers, and so on) – an important component of many scientists' jobs. The aim of this study is to determine how universities and science-based institutions measure and reward staff for such activities. We seek to characterize the differences between (a) the views of employees and their administrators, (b) what is viewed as important and what is rewarded, and (c) what is rewarded and what is measured. These insights will help the Global Young Academy guide best practices in the measurement of "engagement" activities.

**Section 1: How are different activities valued at your institution?**

The next two questions ask you to indicate the level of importance and reward for four different types of activities. Each question pertains to a different focus of the activities: (a) how you perceive your institution to value the different activities, and (b) the social impact/benefit that you perceive associated with each activity.

**Question 1**

PLEASE CONSIDER THE CURRENT **PROCEDURE AT YOUR INSTITUTION** FOR THE FOLLOWING QUESTION

How much do you think your institution **REWARDS** the following activities for the **HIRING**, **PERIODIC REVIEWS**, and **PROMOTIONS** processes? [Respondents could select only one option for each activity/reward combination]

| | **Hiring** | **Periodic Reviews** | **Promotions** |
|---|---|---|---|
| **Research (to the point of publication)** | □ Highly rewarded<br>□ Moderately Rewarded<br>□ Slightly Rewarded<br>□ Not Rewarded<br>□ Unclear | □ Highly rewarded<br>□ Moderately Rewarded<br>□ Slightly Rewarded<br>□ Not Rewarded<br>□ Unclear | □ Highly rewarded<br>□ Moderately Rewarded<br>□ Slightly Rewarded<br>□ Not Rewarded<br>□ Unclear |
| **Internal Service (e.g., graduate program advising)** | □ Highly rewarded<br>□ Moderately Rewarded<br>□ Slightly Rewarded<br>□ Not Rewarded<br>□ Unclear | □ Highly rewarded<br>□ Moderately Rewarded<br>□ Slightly Rewarded<br>□ Not Rewarded<br>□ Unclear | □ Highly rewarded<br>□ Moderately Rewarded<br>□ Slightly Rewarded<br>□ Not Rewarded<br>□ Unclear |
| **Teaching (undergraduate and graduate)** | □ Highly rewarded<br>□ Moderately Rewarded<br>□ Slightly Rewarded<br>□ Not Rewarded<br>□ Unclear | □ Highly rewarded<br>□ Moderately Rewarded<br>□ Slightly Rewarded<br>□ Not Rewarded<br>□ Unclear | □ Highly rewarded<br>□ Moderately Rewarded<br>□ Slightly Rewarded<br>□ Not Rewarded<br>□ Unclear |
| **Engagement: Research Dissemination, Public Education, and External Service (e.g., public engagement, consulting with policymakers)** | □ Highly rewarded<br>□ Moderately Rewarded<br>□ Slightly Rewarded<br>□ Not Rewarded<br>□ Unclear | □ Highly rewarded<br>□ Moderately Rewarded<br>□ Slightly Rewarded<br>□ Not Rewarded<br>□ Unclear | □ Highly rewarded<br>□ Moderately Rewarded<br>□ Slightly Rewarded<br>□ Not Rewarded<br>□ Unclear |

**Question 2**
PLEASE PROVIDE YOUR **PERSONAL VIEW** FOR THE FOLLOWING QUESTION

How much do you think the following activities **HAS SOCIAL IMPACT/BENEFIT**.

| | |
|---|---|
| **Research (to the point of publication)** | ◻ High social impact<br>◻ Moderate social impact<br>◻ Slight social impact<br>◻ No social impact<br>◻ Unclear |
| **Teaching (undergraduate and graduate)** | ◻ High social impact<br>◻ Moderate social impact<br>◻ Slight social impact<br>◻ No social impact<br>◻ Unclear |
| **Internal Service (e.g., graduate program advising** | ◻ High social impact<br>◻ Moderate social impact<br>◻ Slight social impact<br>◻ No social impact<br>◻ Unclear |
| **Engagement: Research Dissemination, Public Education, and External Service (e.g., public engagement, consultation with policymakers)** | ◻ High social impact<br>◻ Moderate social impact<br>◻ Slight social impact<br>◻ No social impact<br>◻ Unclear |

**Section 2: How is Engagement Assessed?**
PLEASE CONSIDER THE CURRENT **PROCEDURE AT YOUR INSTITUTION** FOR THIS SECTION

**Question 3**
What information about engagement is requested by your Institution?

- ◻ Free written responses (i.e., you describe what you do)
- ◻ Established metrics (e.g., hours dedicated, number of engagements, audience reached)
- ◻ Both
- ◻ Neither
- ◻ Don't Know

Please indicate what kind of information is asked of you and how you think it is assessed.

| |
|---|
| |

**Question 4**

Excellence in Engagement can take many forms. Below we list seven aspects of excellence that might be important. Please indicate how much you think your institution rewards engagement among these different dimensions for **hiring, periodic reviews, and promotion**.

| | **Hiring** | **Periodic Reviews** | **Promotion** |
|---|---|---|---|
| **Reach (e.g., size of audience, readership)** | ◻ Highly rewarded<br>◻ Moderately rewarded<br>◻ Slightly rewarded<br>◻ Not rewarded<br>◻ Unclear | ◻ Highly rewarded<br>◻ Moderately rewarded<br>◻ Slightly rewarded<br>◻ Not rewarded<br>◻ Unclear | ◻ Highly rewarded<br>◻ Moderately rewarded<br>◻ Slightly rewarded<br>◻ Not rewarded<br>◻ Unclear |
| **Rigor (the quality of the representation of science)** | ◻ Highly rewarded<br>◻ Moderately rewarded<br>◻ Slightly rewarded<br>◻ Not rewarded<br>◻ Unclear | ◻ Highly rewarded<br>◻ Moderately rewarded<br>◻ Slightly rewarded<br>◻ Not rewarded<br>◻ Unclear | ◻ Highly rewarded<br>◻ Moderately rewarded<br>◻ Slightly rewarded<br>◻ Not rewarded<br>◻ Unclear |
| **Innovation (novelty of the engagement activity)** | ◻ Highly rewarded<br>◻ Moderately rewarded<br>◻ Slightly rewarded<br>◻ Not rewarded<br>◻ Unclear | ◻ Highly rewarded<br>◻ Moderately rewarded<br>◻ Slightly rewarded<br>◻ Not rewarded<br>◻ Unclear | ◻ Highly rewarded<br>◻ Moderately rewarded<br>◻ Slightly rewarded<br>◻ Not rewarded<br>◻ Unclear |
| **Number/Quantity of Efforts** | ◻ Highly rewarded<br>◻ Moderately rewarded<br>◻ Slightly rewarded<br>◻ Not rewarded<br>◻ Unclear | ◻ Highly rewarded<br>◻ Moderately rewarded<br>◻ Slightly rewarded<br>◻ Not rewarded<br>◻ Unclear | ◻ Highly rewarded<br>◻ Moderately rewarded<br>◻ Slightly rewarded<br>◻ Not rewarded<br>◻ Unclear |
| **Depth of Effort (the work behind individual efforts, e.g., writing op-eds versus signing petitions)** | ◻ Highly rewarded<br>◻ Moderately rewarded<br>◻ Slightly rewarded<br>◻ Not rewarded<br>◻ Unclear | ◻ Highly rewarded<br>◻ Moderately rewarded<br>◻ Slightly rewarded<br>◻ Not rewarded<br>◻ Unclear | ◻ Highly rewarded<br>◻ Moderately rewarded<br>◻ Slightly rewarded<br>◻ Not rewarded<br>◻ Unclear |
| **Prominence (e.g., sitting on a board of directors vs. volunteering, giving keynote or invited talks vs. uninvited talks)** | ◻ Highly rewarded<br>◻ Moderately rewarded<br>◻ Slightly rewarded<br>◻ Not rewarded<br>◻ Unclear | ◻ Highly rewarded<br>◻ Moderately rewarded<br>◻ Slightly rewarded<br>◻ Not rewarded<br>◻ Unclear | ◻ Highly rewarded<br>◻ Moderately rewarded<br>◻ Slightly rewarded<br>◻ Not rewarded<br>◻ Unclear |
| **Outcomes (changes as a result of the activity)** | ◻ Highly rewarded<br>◻ Moderately rewarded<br>◻ Slightly rewarded<br>◻ Not rewarded<br>◻ Unclear | ◻ Highly rewarded<br>◻ Moderately rewarded<br>◻ Slightly rewarded<br>◻ Not rewarded<br>◻ Unclear | ◻ Highly rewarded<br>◻ Moderately rewarded<br>◻ Slightly rewarded<br>◻ Not rewarded<br>◻ Unclear |

**Question 5**
Which of these seven dimensions do you consider being covered by any of the metrics/information on engagement used by your institution? Select all that apply.

- ◻ **Reach** (e.g., size of audience, readership)
- ◻ **Rigor** (the quality of science that is reflected in the engagement activity)
- ◻ **Innovation** (novelty of the engagement activity)
- ◻ **Number/Quantity of Efforts**
- ◻ **Depth** of Effort (the work behind individual efforts, e.g., writing op-eds versus signing petitions)
- ◻ **Prominence** (e.g., sitting on a board of directors vs. volunteering, giving keynote or invited talks vs. uninvited talks)
- ◻ **Outcomes** (changes as a result of the activity)

**Question 6**
Which metrics requested by your institution (in question 3) covers these seven dimensions (in question 5)? If no metrics are requested please indicate this

**Section 3: What Outreach Activities Do You Engage in, and What Are You Rewarded for?**

**Question 7**

How often have you participated in the following activities in any capacity during the past year?

| | None | 1-3 | 4-6 | 7+ | Don't know |
|---|---|---|---|---|---|
| **a. Interpreted science for policy makers and the public without taking a policy position (e.g., wrote an article for popular consumption, interviewed by a journalist, given a public seminar, etc…)** | ◻ | ◻ | ◻ | ◻ | ◻ |
| **b. Integrated science into decision-making through collaborations with policy makers without taking a policy position (e.g., provided expert advice, wrote science briefs or white papers, etc…)** | ◻ | ◻ | ◻ | ◻ | ◻ |
| **c. Actively taken a position on a particular issue based on science (e.g., organized campaigns, wrote op-eds, contacted politicians to voice opinions, attended protests, etc…)** | ◻ | ◻ | ◻ | ◻ | ◻ |
| **d. Acted as a decision-maker with regard to policy** | ◻ | ◻ | ◻ | ◻ | ◻ |
| **e. Directly involved communities or stakeholders in research design, execution, and/or knowledge dissemination (participatory research)** | ◻ | ◻ | ◻ | ◻ | ◻ |

**Question 8**
PLEASE CONSIDER THE CURRENT **PROCEDURE AT YOUR INSTITUTION** FOR THE FOLLOWING QUESTION

Do you think you are doing more than is rewarded for by your institution?

| | **Yes** | **No** |
|---|---|---|
| **Interpret Science** | □ | □ |
| **Integrate Science** | □ | □ |
| **Actively take a position** | □ | □ |
| **Act as a decision-maker** | □ | □ |
| **Participatory research** | □ | □ |

**ANSWER THE FOLLOWING IF YOU SELECTED "YES" TO THE CORRESPONDING CATEGORIES ABOVE**
**How much MORE DO YOU FEEL YOU DO than is rewarded?**
**Interpret Science**
0% □
1-25% □
26-50% □
51-100% □
100+% □
**Integrate Science**
0% □
1-25% □
26-50% □
51-100% □
100+% □
**Actively take a position**
0% □
1-25% □
26-50% □
51-100% □
100+% □
**Act as a decision-maker**
0% □
1-25% □
26-50% □
51-100% □
100+% □
**Participatory research**
0% □
1-25% □
26-50% □
51-100% □
100+% □

**ANSWER THE FOLLOWING IF YOU SELECTED "NO" TO THE CORRESPONDING CATEGORIES ABOVE**

**How much MORE THAN YOU CURRENTLY DO would you be rewarded for?**

**Interpret Science**

- 0% ◻
- 1-25% ◻
- 26-50% ◻
- 51-100% ◻
- 100+% ◻

**Integrate Science**

- 0% ◻
- 1-25% ◻
- 26-50% ◻
- 51-100% ◻
- 100+% ◻

**Actively take a position**

- 0% ◻
- 1-25% ◻
- 26-50% ◻
- 51-100% ◻
- 100+% ◻

**Act as a decision-maker**

- 0% ◻
- 1-25% ◻
- 26-50% ◻
- 51-100% ◻
- 100+% ◻

**Participatory research**

- 0% ◻
- 1-25% ◻
- 26-50% ◻
- 51-100% ◻
- 100+% ◻

**ANSWER THE FOLLOWING IF YOU SELECTED "YES" TO THE CORRESPONDING CATEGORIES ABOVE**

**Do you feel that your institution should reward you for your additional activities?**

How should your institution improve the review process for **interpreting science**? Select all that apply

- ◻ More weight given to this kind of activity
- ◻ Additional metrics for this kind of activity
- ◻ Higher expectations for amount of this activity
- ◻ Other (specify) [ ]
- ◻ I don't think my institution should reward me for additional activities

How should your institution improve the review process for **integrating science**? Select all that apply

- ◻ More weight given to this kind of activity
- ◻ Additional metrics for this kind of activity
- ◻ Higher expectations for amount of this activity
- ◻ Other (specify) [ ]
- ◻ I don't think my institution should reward me for additional activities

How should your institution improve the review process for **actively taking a position**? Select all that apply

- ◻ More weight given to this kind of activity
- ◻ Additional metrics for this kind of activity
- ◻ Higher expectations for amount of this activity
- ◻ Other (specify) [ ]
- ◻ I don't think my institution should reward me for additional activities

How should your institution improve the review process for **acting as a decision maker**? Select all that apply

- ◻ More weight given to this kind of activity
- ◻ Additional metrics for this kind of activity
- ◻ Higher expectations for amount of this activity
- ◻ Other (specify) [ ]
- ◻ I don't think my institution should reward me for additional activities

How should your institution improve the review process for **participatory research**? Select all that apply

- ◻ More weight given to this kind of activity
- ◻ Additional metrics for this kind of activity
- ◻ Higher expectations for amount of this activity
- ◻ Other (specify) [ ]
- ◻ I don't think my institution should reward me for additional activities

**Question 9.**

Please indicate your agreement with the following statements

| | |
|---|---|
| **I engage to fulfill a sense of social responsibility** | □ Strongly Disagree<br>□ Disagree<br>□ Neutral<br>□ Agree<br>□ Strongly Agree |
| **I engage to contribute to a better world** | □ Strongly Disagree<br>□ Disagree<br>□ Neutral<br>□ Agree<br>□ Strongly Agree |
| **I engage because it can lead to career benefits** | □ Strongly Disagree<br>□ Disagree<br>□ Neutral<br>□ Agree<br>□ Strongly Agree |
| **I engage to help develop transferable skills in communication** | □ Strongly Disagree<br>□ Disagree<br>□ Neutral<br>□ Agree<br>□ Strongly Agree |
| **I engage to excite the public about science and/or the humanities** | □ Strongly Disagree<br>□ Disagree<br>□ Neutral<br>□ Agree<br>□ Strongly Agree |
| **I engage to confront misunderstandings about science and/or the humanities** | □ Strongly Disagree<br>□ Disagree<br>□ Neutral<br>□ Agree<br>□ Strongly Agree |
| **I engage to build public understanding and/or trust in science** | □ Strongly Disagree<br>□ Disagree<br>□ Neutral<br>□ Agree<br>□ Strongly Agree |
| **I engage to raise my status as a research personality** | □ Strongly Disagree<br>□ Disagree<br>□ Neutral<br>□ Agree<br>□ Strongly Agree |
| **I engage to combat poor or ideological policymaking** | □ Strongly Disagree<br>□ Disagree<br>□ Neutral<br>□ Agree<br>□ Strongly Agree |

| | |
|---|---|
| **I am not motivated to engage** | ◻ Strongly Disagree<br>◻ Disagree<br>◻ Neutral<br>◻ Agree<br>◻ Strongly Agree |

**Section 4: Demographics**

**Question 10**
What is/are your affiliation(s)? Specifically (if applicable), what institution and department are you a part of?

**Question 11**
What is your title?

**Question 12**
What type of organization do you serve in?

- ◻ Academic
- ◻ Agency (federal/provincial/state)
- ◻ Non-Governmental Organization
- ◻ Private Company
- ◻ Other (please specify)

**ANSWER THE FOLLOWING IF YOU SELECTED "ACADEMIC" TO THE QUESTION ABOVE**
**Within academia, please indicate your core job responsibilities. Select as many options as applicable.**

◻ Research ◻ Teaching ◻ Administration (e.g. department head, dean) ◻ Outreach/ Engagement

**ANSWER THE FOLLOWING IF YOU SELECTED "AGENCY" TO THE QUESTION ABOVE**
**Within a government agency, please indicate your core job responsibilities. Select as many options as applicable.**

◻ Research ◻ Policy/ Resource Management ◻ Administration/ Personnel management ◻ Outreach/ Engagement

**ANSWER THE FOLLOWING IF YOU SELECTED "NON-GOVERNMENTAL ORGANIZATION" TO THE QUESTION ABOVE**
**Within an NGO, please indicate your core job responsibilities. Select as many options as applicable.**

◻ Research ◻ Policy analysis/ Advocacy ◻ Administration/ Personnel management ◻ Outreach/ Engagement

**ANSWER THE FOLLOWING IF YOU SELECTED "PRIVATE COMPANY" TO THE QUESTION ABOVE**
**Within a private company, please indicate your core job responsibilities. Select as many options as applicable.**

◻ Research ◻ Business/ Operations ◻ Administration/ Personnel management ◻ Outreach/ Engagement

**Question 13**
Which of the following do you identify with the most?

- ◻ Natural sciences
- ◻ Physical sciences
- ◻ Health sciences
- ◻ Applied sciences/engineering
- ◻ Social sciences
- ◻ Humanities
- ◻ Interdisciplinary
- ◻ Other

**Question 14**
Age

◻ <30 ◻ 30-39 ◻ 40-49 ◻ 50-75

**Question 15**
Gender

◻ Male ◻ Female ◻ Other

**Question 16**
Highest degree earned

**Question 17**
Year of completion of degree

**Question 18**
Have you ever served on a review committee for hiring, periodic review, or promotion for a fellow scientist or scholar?

◻ Yes ◻ No

**ANSWER THE FOLLOWING IF YOU SELECTED "YES" TO THE QUESTION ABOVE**
**Which of the following were included in the review committee(s) you've served on? Select as many options as applicable**

◻ Hiring ◻ Periodic review ◻ Promotion

**Question 19**
Do you currently oversee review committees, as Department Head or Dean, or in a similar position?

◻ Yes ◻ No

**Question 20**
If you are university or college faculty, are you tenured?

◻ Yes ◻ No ◻ Not applicable

**ANSWER THE FOLLOWING IF YOU SELECTED "YES" TO THE QUESTION ABOVE**
How recently did you acquire tenure?

◻ 0-1 years ago ◻ 2-5 years ago ◻ 5+ years ago

**ANSWER THE FOLLOWING IF YOU SELECTED "NO" TO THE QUESTION ABOVE**
How soon will you go up for tenure?

◻ 0-1 years ago ◻ 2-5 years ago ◻ 5+ years ago

**Comments**

## Part 2: Survey sent out to students

## Scientists and Engagement/Outreach

There are relatively well developed metrics for some research and teaching activities of scientists (scientists interpreted most broadly to include scholars of all kinds), but other activities have received relatively less attention. This study focuses on engagement or outreach — communicating or working with people outside of research institutions (e.g., journalists, industry, policy-makers etc.)—an important component of many scientists' jobs. The aim of this study is to determine how universities and science-based institutions measure and reward staff for such activities. We seek to characterize the differences between (a) the views of students and their supervisors, (b) what is viewed as important and what is rewarded, and (c) what is rewarded and what is measured. These insights will help the Global Young Academy guide best-practices in the measurement of 'engagement' activities.

## Section 1: How are different activities valued?

The next two questions ask you to indicate the level of importance and reward for four different types of activities in three contexts: **funding applications** (to your most relevant funder), **professional opportunities** (in your current expected career trajectory), and your **supervisory committee's overall view** of you (e.g., in reference letters). Each question pertains to a different focus of the activities: (a) how you perceive your institution to value the different activities, and (b) the social impact/benefit that you perceive associated with each activity.

**Question 1**

PLEASE CONSIDER THE CURRENT **PROCEDURE AT YOUR INSTITUTION** FOR THE FOLLOWING QUESTION

How much do you think your institution **REWARDS** the following activities for **FUNDING APPLICATIONS**, **PROFESSIONAL OPPORTUNITIES**, and **SUPERVISORY ASSESSMENTS**?

[Respondents could select only one option for each activity/reward combination]

| | **Funding Applications** | **Professional Opportunities** | **Supervisory Assessments** |
|---|---|---|---|
| **Research** (to the point of publication) | ☐ Highly rewarded<br>☐ Moderately Rewarded<br>☐ Slightly Rewarded<br>☐ Not Rewarded<br>☐ Unclear | ☐ Highly rewarded<br>☐ Moderately Rewarded<br>☐ Slightly Rewarded<br>☐ Not Rewarded<br>☐ Unclear | ☐ Highly rewarded<br>☐ Moderately Rewarded<br>☐ Slightly Rewarded<br>☐ Not Rewarded<br>☐ Unclear |
| **Teaching** (e.g., TA-ing) | ☐ Highly rewarded<br>☐ Moderately Rewarded<br>☐ Slightly Rewarded<br>☐ Not Rewarded<br>☐ Unclear | ☐ Highly rewarded<br>☐ Moderately Rewarded<br>☐ Slightly Rewarded<br>☐ Not Rewarded<br>☐ Unclear | ☐ Highly rewarded<br>☐ Moderately Rewarded<br>☐ Slightly Rewarded<br>☐ Not Rewarded<br>☐ Unclear |
| **Internal Service** (e.g., student government, university committees, academic meeting organization) | ☐ Highly rewarded<br>☐ Moderately Rewarded<br>☐ Slightly Rewarded<br>☐ Not Rewarded<br>☐ Unclear | ☐ Highly rewarded<br>☐ Moderately Rewarded<br>☐ Slightly Rewarded<br>☐ Not Rewarded<br>☐ Unclear | ☐ Highly rewarded<br>☐ Moderately Rewarded<br>☐ Slightly Rewarded<br>☐ Not Rewarded<br>☐ Unclear |
| **Engagement**: Research Dissemination, Public Education, and External Service (e.g., public engagement, consulting with policymakers) | ☐ Highly rewarded<br>☐ Moderately Rewarded<br>☐ Slightly Rewarded<br>☐ Not Rewarded<br>☐ Unclear | ☐ Highly rewarded<br>☐ Moderately Rewarded<br>☐ Slightly Rewarded<br>☐ Not Rewarded<br>☐ Unclear | ☐ Highly rewarded<br>☐ Moderately Rewarded<br>☐ Slightly Rewarded<br>☐ Not Rewarded<br>☐ Unclear |

**Question 2**

PLEASE PROVIDE YOUR **PERSONAL VIEW** FOR THE FOLLOWING QUESTION

How much do you think the following activities **HAS SOCIAL IMPACT/BENEFIT**.

| | |
|---|---|
| **Research** (to the point of publication) | ☐ High social impact<br>☐ Moderate social impact<br>☐ Slight social impact<br>☐ No social impact<br>☐ Unclear |
| **Teaching** (e.g. TAing) | ☐ High social impact<br>☐ Moderate social impact<br>☐ Slight social impact<br>☐ No social impact<br>☐ Unclear |
| **Internal Service** (e.g., student government, university committees, academic meeting organization) | ☐ High social impact<br>☐ Moderate social impact<br>☐ Slight social impact<br>☐ No social impact<br>☐ Unclear |
| **Engagement**: Research Dissemination, Public Education, and External Service (e.g., public engagement, consultation with policymakers) | ☐ High social impact<br>☐ Moderate social impact<br>☐ Slight social impact<br>☐ No social impact<br>☐ Unclear |

## Section 2: How is Engagement Assessed?

PLEASE CONSIDER THE CURRENT **PROCEDURE AT YOUR INSTITUTION** FOR THIS SECTION

**Question 3**

Does your institution keep track of your engagement? What information about engagement is requested by your Institution?

- ☐ Free written responses (i.e., you describe what you do)
- ☐ Established metrics (e.g., hours dedicated, number of engagements, audience reached)
- ☐ Both
- ☐ Neither
- ☐ Don't Know

Please indicate what kind of information is asked of you and how you think it is assessed.

**Question 4**

Excellence in Engagement can take many forms. Below we list seven aspects of excellence that might be important. Please indicate how much you think your institution rewards engagement among these different dimensions for **funding applications, professional opportunities, and supervisory assessments** (e.g., how it contributes to a reference letter).

| | Funding Applications | Professional Opportunities | Supervisory Assessments |
|---|---|---|---|
| **Reach** (e.g., size of audience, readership) | ☐ Highly rewarded<br>☐ Moderately rewarded<br>☐ Slightly rewarded<br>☐ Not rewarded<br>☐ Unclear | ☐ Highly rewarded<br>☐ Moderately rewarded<br>☐ Slightly rewarded<br>☐ Not rewarded<br>☐ Unclear | ☐ Highly rewarded<br>☐ Moderately rewarded<br>☐ Slightly rewarded<br>☐ Not rewarded<br>☐ Unclear |
| **Rigor** (the quality of the representation of science) | ☐ Highly rewarded<br>☐ Moderately rewarded<br>☐ Slightly rewarded<br>☐ Not rewarded<br>☐ Unclear | ☐ Highly rewarded<br>☐ Moderately rewarded<br>☐ Slightly rewarded<br>☐ Not rewarded<br>☐ Unclear | ☐ Highly rewarded<br>☐ Moderately rewarded<br>☐ Slightly rewarded<br>☐ Not rewarded<br>☐ Unclear |
| **Innovation** (novelty of the engagement activity) | ☐ Highly rewarded<br>☐ Moderately rewarded<br>☐ Slightly rewarded<br>☐ Not rewarded<br>☐ Unclear | ☐ Highly rewarded<br>☐ Moderately rewarded<br>☐ Slightly rewarded<br>☐ Not rewarded<br>☐ Unclear | ☐ Highly rewarded<br>☐ Moderately rewarded<br>☐ Slightly rewarded<br>☐ Not rewarded<br>☐ Unclear |
| **Number/Quantity of Efforts** | ☐ Highly rewarded<br>☐ Moderately rewarded<br>☐ Slightly rewarded<br>☐ Not rewarded<br>☐ Unclear | ☐ Highly rewarded<br>☐ Moderately rewarded<br>☐ Slightly rewarded<br>☐ Not rewarded<br>☐ Unclear | ☐ Highly rewarded<br>☐ Moderately rewarded<br>☐ Slightly rewarded<br>☐ Not rewarded<br>☐ Unclear |
| **Depth of Effort** (the work behind individual efforts, e.g., writing op-eds versus signing petitions) | ☐ Highly rewarded<br>☐ Moderately rewarded<br>☐ Slightly rewarded<br>☐ Not rewarded<br>☐ Unclear | ☐ Highly rewarded<br>☐ Moderately rewarded<br>☐ Slightly rewarded<br>☐ Not rewarded<br>☐ Unclear | ☐ Highly rewarded<br>☐ Moderately rewarded<br>☐ Slightly rewarded<br>☐ Not rewarded<br>☐ Unclear |
| **Prominence** (e.g., sitting on a board of directors vs. volunteering, giving keynote or invited talks vs. uninvited talks) | ☐ Highly rewarded<br>☐ Moderately rewarded<br>☐ Slightly rewarded<br>☐ Not rewarded<br>☐ Unclear | ☐ Highly rewarded<br>☐ Moderately rewarded<br>☐ Slightly rewarded<br>☐ Not rewarded<br>☐ Unclear | ☐ Highly rewarded<br>☐ Moderately rewarded<br>☐ Slightly rewarded<br>☐ Not rewarded<br>☐ Unclear |
| **Outcomes** (changes as a result of the activity) | ☐ Highly rewarded<br>☐ Moderately rewarded<br>☐ Slightly rewarded<br>☐ Not rewarded<br>☐ Unclear | ☐ Highly rewarded<br>☐ Moderately rewarded<br>☐ Slightly rewarded<br>☐ Not rewarded<br>☐ Unclear | ☐ Highly rewarded<br>☐ Moderately rewarded<br>☐ Slightly rewarded<br>☐ Not rewarded<br>☐ Unclear |

**Question 5**

Which of these seven dimensions do you consider to be covered by any of the metrics/information on engagement used by the relevant institutions/individuals? Select all that apply.

- ☐ **Reach** (e.g., size of audience, readership)
- ☐ **Rigor** (the quality of science that is reflected in the engagement activity)
- ☐ **Innovation** (novelty of the engagement activity)
- ☐ **Number/Quantity of Efforts**
- ☐ **Depth** of Effort (the work behind individual efforts, e.g., writing op-eds versus signing petitions)
- ☐ **Prominence** (e.g., sitting on a board of directors vs. volunteering, giving keynote or invited talks vs. uninvited talks)
- ☐ **Outcomes** (changes as a result of the activity)
- ☐ **None**

**Question 6**

Which metrics requested by your institution (in question 3) covers these seven dimensions (in question 5)? If no metrics are requested please indicate this

| |
|---|
| |

## Section 3: What Outreach Activities Do You Engage in, and What Are You Rewarded for?

### Question 7

How often have you participated in the following activities in any capacity during the past year?

| | None | 1-3 | 4-6 | 7+ | Don't know |
|---|---|---|---|---|---|
| **a. Interpreted science for policy makers and the public without taking a policy position** (e.g., wrote an article for popular consumption, interviewed by a journalist, given a public seminar, etc…) | ☐ | ☐ | ☐ | ☐ | ☐ |
| **b. Integrated science into decision-making through collaborations with policy makers without taking a policy position** (e.g., provided expert advice, wrote science briefs or white papers, etc…) | ☐ | ☐ | ☐ | ☐ | ☐ |
| **c. Actively taken a position on a particular issue based on science** (e.g., organized campaigns, wrote op-eds, contacted politicians to voice opinions, attended protests, etc…) | ☐ | ☐ | ☐ | ☐ | ☐ |
| **d. Acted as a decision-maker with regard to policy** | ☐ | ☐ | ☐ | ☐ | ☐ |
| **e. Directly involved communities or stakeholders in research design, execution, and/or knowledge dissemination** (participatory research) | ☐ | ☐ | ☐ | ☐ | ☐ |

**Question 8**

Do you think you are doing more than is rewarded for by the relevant institutions/individuals?

| | Yes | No |
|---|---|---|
| **Interpret Science** | ◻ | ◻ |
| **Integrate Science** | ◻ | ◻ |
| **Actively take a position** | ◻ | ◻ |
| **Act as a decision-maker** | ◻ | ◻ |
| **Participatory research** | ◻ | ◻ |

**ANSWER THE FOLLOWING IF YOU SELECTED "YES" TO THE CORRESPONDING CATEGORIES ABOVE**

**How much MORE DO YOU FEEL YOU DO than is rewarded?**

**Interpret Science**

0% ◻
1-25% ◻
26-50% ◻
51-100% ◻
100+% ◻

**Integrate Science**

0% ◻
1-25% ◻
26-50% ◻
51-100% ◻
100+% ◻

**Actively take a position**

0% ◻
1-25% ◻
26-50% ◻
51-100% ◻
100+% ◻

**Act as a decision-maker**

0% ◻
1-25% ◻
26-50% ◻
51-100% ◻

100+% ☐

**Participatory research**

0% ☐
1-25% ☐
26-50% ☐
51-100% ☐
100+% ☐

**ANSWER THE FOLLOWING IF YOU SELECTED “NO” TO THE CORRESPONDING CATEGORIES ABOVE**

**How much MORE THAN YOU CURRENTLY DO would you be rewarded for?**

**Interpret Science**

0% ☐
1-25% ☐
26-50% ☐
51-100% ☐
100+% ☐

**Integrate Science**

0% ☐
1-25% ☐
26-50% ☐
51-100% ☐
100+% ☐

**Actively take a position**

0% ☐
1-25% ☐
26-50% ☐
51-100% ☐
100+% ☐

**Act as a decision-maker**

0% ☐
1-25% ☐
26-50% ☐
51-100% ☐
100+% ☐

**Participatory research**

| | |
|---|---|
| 0% | ☐ |
| 1-25% | ☐ |
| 26-50% | ☐ |
| 51-100% | ☐ |
| 100+% | ☐ |

**ANSWER THE FOLLOWING IF YOU SELECTED "YES" TO THE CORRESPONDING CATEGORIES ABOVE**

**Do you feel that relevant institutions/individuals should reward you for your additional activities?**

How should your institution improve the review process for **interpreting science**? Select all that apply

- ☐ More weight given to this kind of activity
- ☐ Additional metrics for this kind of activity
- ☐ Higher expectations for amount of this activity
- ☐ Other (specify) [ ]
- ☐ I don't think I should be rewarded for additional activities

How should your institution improve the review process for **integrating science**? Select all that apply

- ☐ More weight given to this kind of activity
- ☐ Additional metrics for this kind of activity
- ☐ Higher expectations for amount of this activity
- ☐ Other (specify) [ ]
- ☐ I don't think I should be rewarded for additional activities

How should your institution improve the review process for **actively taking a position**? Select all that apply

- ☐ More weight given to this kind of activity
- ☐ Additional metrics for this kind of activity
- ☐ Higher expectations for amount of this activity
- ☐ Other (specify) [ ]
- ☐ I don't think I should be rewarded for additional activities

How should your institution improve the review process for **acting as a decision maker**? Select all that apply

- ☐ More weight given to this kind of activity
- ☐ Additional metrics for this kind of activity
- ☐ Higher expectations for amount of this activity
- ☐ Other (specify) [ ]
- ☐ I don't think I should be rewarded for additional activities

How should your institution improve the review process for **participatory research**? Select all that apply

- ☐ More weight given to this kind of activity

- ☐ Additional metrics for this kind of activity
- ☐ Higher expectations for amount of this activity
- ☐ Other (specify) ________
- ☐ I don't think I should be rewarded for additional activities

**Question 9.**

Please indicate your agreement with the following statements

| | |
|---|---|
| **I engage to fulfill a sense of social responsibility** | ◻ Strongly Disagree<br>◻ Disagree<br>◻ Neutral<br>◻ Agree<br>◻ Strongly Agree |
| **I engage to contribute to a better world** | ◻ Strongly Disagree<br>◻ Disagree<br>◻ Neutral<br>◻ Agree<br>◻ Strongly Agree |
| **I engage because it can lead to career benefits** | ◻ Strongly Disagree<br>◻ Disagree<br>◻ Neutral<br>◻ Agree<br>◻ Strongly Agree |
| **I engage to help develop transferable skills in communication** | ◻ Strongly Disagree<br>◻ Disagree<br>◻ Neutral<br>◻ Agree<br>◻ Strongly Agree |
| **I engage to excite the public about science and/or the humanities** | ◻ Strongly Disagree<br>◻ Disagree<br>◻ Neutral<br>◻ Agree<br>◻ Strongly Agree |
| **I engage to confront misunderstandings about science and/or the humanities** | ◻ Strongly Disagree<br>◻ Disagree<br>◻ Neutral<br>◻ Agree<br>◻ Strongly Agree |
| **I engage to build public understanding and/or trust in science** | ◻ Strongly Disagree<br>◻ Disagree<br>◻ Neutral<br>◻ Agree<br>◻ Strongly Agree |
| **I engage to raise my status as a research personality** | ◻ Strongly Disagree<br>◻ Disagree<br>◻ Neutral<br>◻ Agree<br>◻ Strongly Agree |
| **I engage to combat poor or ideological policymaking** | ◻ Strongly Disagree<br>◻ Disagree<br>◻ Neutral<br>◻ Agree<br>◻ Strongly Agree |

| | |
|---|---|
| **I am not motivated to engage** | ◻ Strongly Disagree<br>◻ Disagree<br>◻ Neutral<br>◻ Agree<br>◻ Strongly Agree |

## Section 4: Demographics

**Question 10**

What is/are your affiliation(s)? Specifically (if applicable), what institution and department are you a part of?

**Question 11**

What is your title?

**Question 12**

What funding council(s) do you apply to?

**Question 13**

What is your intended career?

- ☐ Academic
- ☐ Agency (federal/provincial/state)
- ☐ Non-Governmental Organization
- ☐ Private Company
- ☐ Other (please specify)

**Question 14**

Which of the following do you identify with the most?

- ☐ Natural sciences
- ☐ Physical sciences
- ☐ Health sciences
- ☐ Applied sciences/engineering
- ☐ Social sciences
- ☐ Humanities
- ☐ Interdisciplinary
- ☐ Other

**Question 15**

Age

☐ <30 ☐ 30-40 ☐ 40-50 ☐ 50-75

**Question 16**

Gender

☐ Male ☐ Female ☐ Other

**Question 17**

Highest degree earned

**Question 18**

Year of completion of degree

## Comments
