# Supplementary material for: Researcher engagement in policy deemed societally beneficial yet unrewarded
Source: Front Ecol Environ. 2019 Jul 30;17(7):375–82. doi: 10.1002/fee.2084 (PMC6910643; doi:10.1002/fee.2084)
Supplement: Supplementary file 8 — WebPanel 2 [file FEE-17-375-s008.pdf]

WebPanel 2. Supporting methods for exploring sampling bias, using model averaging methods to determine which motivations were most important in predicting activity levels, determining geographic and disciplinary consistency in responses, and discussing the treatment of ordinal data

Considering sampling bias

Because we relied partly on a snowball sampling approach, we risked a sampling bias that was in favor of engagement. We tested for pro-engagement bias in the survey by first comparing the proportion of our survey participants' research institutions that mention at least one of engagement, social service, and/or public dissemination in their mission statements, stated values, and/or organizational strategies (found on institutional websites) to a random sample of 50 universities around the world. To generate our random sample, we collected a list of global universities from www.timeshighereducation.com and used a random number generator to select 50 universities from this list (Panel Table 1). We found a near-identical proportion (81% versus 82%). Second, we compared responses in perceived institutional reward, societal benefit, perceptions on whether researchers were doing more engagement than their institutions rewarded for, and motivations to engage among members of the Leopold Leadership Program and the Global Young Academy (all members join because of their desires to engage with policy and the public) with all other respondents. We found all responses nearly identical in their response patterns, indicating that respondents with known pro-engagement views did not bias the sample. While this might also indicate that all respondents have pro-engagement bias and does not reflect the larger researcher community, we note that our large sample may buffer against extreme responses, but that even a biased sample can showcase a subpopulation of pro-engagement researchers in institutions. If institutions are not satisfying their motivations that means an entire group of researchers may be underserved by their institutions, especially if those institutions have pro-engagement mission statements.

Model averaging methods

To aid in interpreting the resulting regression coefficients of the model averaging, we put all explanatory variables on a common scale by dividing each by two standard deviations (Gelman 2008). Model averaging is an information theoretic approach (comparing models based on how well they account for information in the data) that examines an exhaustive set of models that can be constructed given the independent variables identified, and considers the fit of each model to the data (Burnham and Anderson 1998). For each model, we used ordinary least squares multiple regression and calculated Akaike weights based on the small sample size corrected Akaike's Information Criterion (AICc), a metric that balances model fit and complexity; these weights were used to calculate model-averaged coefficients for each variable. For each engagement activity for both established researcher and students, we explored all possible 2048 candidate models to generate the model-averaged coefficients. Where predictor variables (stated motivations) had strong support (the 95% confidence of model-averaged coefficients did not cross 0), we discuss the relationship between motivations and levels of engagement behavior. We assessed the explanatory power of our model-averaged results by comparing the rank of a null model (with no independent variables) against our top-ranked models (Singh *et al*. 2014). All models except for models predicting the frequency of students acting as a decision maker were found to have high explanatory power, as null models were ranked very low in every other engagement category for established researchers and students (WebTables 4 and 5). The low explanatory power for models of students acting as a decision maker likely reflect that very few students indicated that they acted as a decision maker at all.

Geographical and disciplinary consistency
We examined differences in responses among geographies (North America, Latin America, Europe, Central Asia, North Africa and the Middle East, East and South Asia, sub-Saharan Africa, and Australia and New Zealand) and disciplines within established researchers and students, and found that results were surprisingly consistent among these classes. We also found that responses were mostly consistent between established researchers and students, though we chose to report these results separately because of the different experiences the two groups have and the different implications the results have between these two groups. We further examined differences regarding motivations between early-career and late-career researchers (pre- and post-tenure) and between students aiming for an academic career and those aiming elsewhere.

The treatment of ordinal data
Much of the data collected from the surveys are ordinal – meaning that the data are directional but categorical. This is different than interval data (integers measured along a continuum) or generally continuous data where the distance value between points in a scale is equal. Ordinal data can have categories with variable inter-category differences. Despite these differences, studies across the natural and social sciences treat ordinal data as if it were continuous. This practice of using continuous analysis for ordinal data has been historically contentious (Knapp 1990) and is argued to this day. A common type of ordinal data, sometimes called "Likert" style data, is commonly used to measure attitudes and perceptions in social and medical sciences (Norman 2010), and similar ordinal scales are used in risk assessments in ecological studies (Murray *et al*. 2016), typically with 5–7 ordered response categories. Researchers who have challenged the use of Likert-style data in analysis commonly intended for continuous data argue that the intervals between Likert values cannot be presumed to be equal, and researchers who rely on statistical techniques based on estimating the effect of this interval (such as regression analysis) can reach dubious conclusions when using Likert-style data (Jamieson 2004). Furthermore, some researchers argue that because of the ordinal scales, reporting on the mean and standard deviation/standard error is inappropriate because the arithmetical functions involved in these calculations assume a continuous scale, and the inevitable fraction that results (eg mean of 3.4) can be uninterpretable when categories are represented by whole numbers (Kuzon *et al*. 1996; Jamieson 2004). For example, if 3 = "fair" and 4 = "good", then what does 3.4 mean?

On the other side are researchers who argue that Likert-style data can be practically analyzed using methods commonly reserved for truly continuous data. First, some authors argue that the phenomena that Likert-style data often measure exist on a continuum, of which the Likert data are trying to capture (Carifio and Perla 2007). Other authors, concerned with the practical over the theoretical aspects of analysis, point out that Likert-style data can act as if they are continuous data if reasonably distributed (Gaito 1980; Carifio and Perla 2007). This argument has been statistically explored by showing that there is less bias in correlation and linear regression using Likert-style data with a greater number of categories, with the biggest drop in bias occurring at 4 categories (Owuor 2001). That is, 4 or more Likert categories can produce significantly less bias in regression analysis than if fewer than 4 Likert categories are used. Still other authors show that many statistical techniques are robust to the challenges that Likert data present, such as demonstrating that F tests are robust to data skewedness and violations of the interval data assumptions (Glass *et al*. 1972). The robustness of tests of central tendency (such as ANOVA) and correlation and regression has been repeated, even in cases where ordinal scales explicitly do not have equal inter-category distances (Norman 2010).

We are not confident in settling this debate, but instead analyze our data in the context of the debate. We attempt to analyze the data being reflective of the potential pitfalls of ordinal data

analysis, but also acknowledge the robustness of statistical tests. This means that while we represent most of our ordinal data as ordinal in representation (eg Figures 2 and 3), we are also comfortable comparing ordinal data through robust tests of central tendency (eg WebTables 1 and 2). We also do treat ordinal responses as continuous in showing aggregate responses (notably, Figure 5), for ease of interpretation and to show the direction of responses (to show if respondents generally agree or disagree with statements). We are careful to focus on the aggregate direction of responses here and not the specific magnitude or score of aggregate responses when interpreting our findings, given the uncertainty that specific measures using Likert-style data can present.

Finally, we do use Likert-style data in linear regression models, being careful of three things. First, we use Likert-style data with greater than 4 category responses (minimizing bias produced by Likert-style data in regression). Second, we attempt to mitigate some issues of variable inter-category distances by rescaling the data according to Gelman (2008). The rescaling technique that Gelman (2008) developed converts all data to scales comparable to a binary variable (0–1); so instead of investigating and interpreting the consequences of moving from one category to another (where the distance between any two can vary), we investigate the consequences of moving from one extreme of our data to the other. In fact, Gelman (2008) shows this technique to be effective across a wide variety of variables, including ordinal variables, in a multiple linear regression. Finally, we again try to limit the interpretation of our results to relate to the direction and relative importance of variables, and do not reach conclusions that rely on more precise measurements (such as the specific coefficient values and their interpretation).

WebReferences

Burnham KP and Anderson DR. 1998. Model selection and inference: a practical information–theoretical approach. New York, NY: Springer-Verlag.

Carifio J and Perla RJ. 2007. Ten common misunderstandings, misconceptions, persistent myths and urban legends about Likert scales and Likert response formats and their antidotes. *J Soc Sci* **3**: 106–16.

Gaito J. 1980. Measurement scales and statistics: resurgence of an old misconception. *Psychol Bull* **87**: 564–67.

Gelman A. 2008. Scaling regression inputs by dividing by two standard deviations. *Stat Med* **27**: 2865–73.

Glass GV, Peckham PD, and Sanders JR. 1972. Consequences of failure to meet assumptions underlying the analyses of variance and covariance. *Rev Educ Res* **42**: 237–88.

Jamieson S. 2004. Likert scales: how to (ab)use them. *Med Educ* **38**: 1212–18.

Knapp TR. 1990. Treating ordinal scales as interval scales: an attempt to resolve the controversy. *Nurs Res* **39**: 121–23.

Kuzon WM, Urbancheck MG, and McCabe S. 1996. The seven deadly sins of statistical analysis. *Ann Plas Surg* **37**: 265–72.

Murray CC, Mach ME, Martone RG, *et al*. 2016. Supporting risk assessment: accounting for indirect risk to ecosystem components. *PLoS ONE* **11**: e0162932.

Norman G. 2010. Likert scales, levels of measurement and the "laws" of statistics. *Adv Health Sci Educ* **15**: 625–32.

Owuor CO. 2001. Implications of using Likert data in multiple regression analysis. PhD dissertation. University of British Columbia.

Singh GG, Tam J, Sisk TD, *et al*. 2014. A more social science: barriers and incentives for scientists engaging in policy. *Front Ecol Environ* **12**: 161–66.

Panel Table 1. Randomly chosen universities and their institutional visions and mission statements

University	Mission of engagement?	Relevant text from institutional vision (including engagement wording when applicable)
Clemson University	yes	extended public service
Central South University	yes	school motto: create knowledge and serve society
University of Illinois at Urbana Champaign	yes	across the nation and around the world through our leadership in learning, discovery, engagement and economic development
Shibaura Institute of Technology Tokyo	yes	Nurturing engineers who learn from society and contribute to society
Paris-Sorbonne University – Paris 4	no	The Sorbonne incites its students to think freely, to construct their own judgment so that they can become responsible and inventive citizens who can promote dignity and a culture of peace
Toyohashi University of Technology	yes	enhance collaboration with the local community
University of Portsmouth	yes	being a proud part of Portsmouth and our region, working in partnership to support and influence the economic, educational, social and cultural life of the city
Niigata University	yes	and collaborative activities between industry, government and academia
Georgetown University	yes	civic and community outreach
University of South Florida	yes	Partnerships to build significant locally- and globally-integrated university-community collaborations through sound scholarly and artistic activities and technological innovation
National Tsing Hua University	no	pursuing knowledge, meticulously nurturing youth and strongly devoting to enrich our society in particular and humanity in general
Panjab University	yes	to emerge as an institution at the pinnacle in innovative teaching, research and community outreach
Hanyang University	yes	four virtues of diligence, honesty, humility and service
Chalmers University of Technology	yes	Chalmers is an active and committed member of the community. We drive key issues and assume responsibility for areas in which we can contribute or where we have a particular role to play as an independent body. As a key partner to industry and society, our contribution is universally acknowledged
University of Notre Dame	yes	Notre Dame as a place of teaching and research, of scholarship and publication, of service and community
New Mexico State University	yes	New Mexico State University is the state's land-grant university, serving the educational needs of New Mexico's diverse population through comprehensive programs of education, research, extension education, and public service

Kwansei Gakuin University	yes	render some useful service to humanity in order that the world may be better for our having lived in it
University of California, Irvine	yes	We will mobilize knowledge, we will serve the people, and we will educate the best and brightest
Sharif University of Technology	no	The fundamental aims of Sharif University of Technology are three. The first is the creation of an institution where students can be instructed in both theoretical and applied sciences with special emphasis on the particular needs of our society. The second is to teach students the advanced knowledge and techniques required to participate in the fields of engineering and technology and to cultivate them into creative engineers, good scientists and innovative technologists. The third is to educate engineers who are ready to be employed, who contribute significantly to their jobs, who have a strong sense of public responsibility and a desire to continue to learn. Particular emphasis is placed on the promotion of multi-disciplinary research at the graduate and doctoral levels
University of Talca	yes	This Plan is structured on the basis of four strategic development focuses, which in turn guide a set of objectives and specific actions on undergraduate, postgraduate, specialties and continuing education training; growth of scientific research; innovation and technology transfer; strengthening community outreach and the generation of distinctive value through administrative management
University of Electronic Science and Technology of China	no	With major national demands as its driving force, it will strive for academic excellence and brave into new frontiers
University of Chemistry and Technology, Prague	no	UCT Prague is a public higher education university pursuing scientific, research, development, and implementation activities
Örebro University	yes	Örebro University cooperates with industry and commerce, local and regional governments and other organisations, both nationally and internationally
University of Salerno	yes	the aim of creating, enriching and, at the same time, offering a scientific, cultural heritage to students, enterprises, institutions and in general to the whole community
Federal University of Ouro Preto	yes	promotes the transformative interaction between University and other sectors of society
Tokyo University of Agriculture and Technology	yes	TUAT contributes to the Japanese advancement of science and technology by promoting cooperation and exchange with research institutions, industries and local communities
University of Haifa	yes	To conduct non-academic activities on condition that these academic activities are in a significant manner its major activity both so far as its goals and the scope of its economic activity are concerned

Federal University of Ceará (UFC)	yes	Within the scope of the Federal University of Ceará extension actions are developed in the following thematic areas: Communication, Culture, Human Rights, Education, Environment, Health, Technology and Labor. The UFC carries out extension actions in the form of programs, projects, courses, events and service rendering
Chang Gung University	yes	Looking ahead, Chang Gung University will continue pursue improvement and enhancement in teaching, research, industry-academic cooperation and international exchange
ITMO University	no	To generate cutting-edge knowledge, implement innovative findings and prepare an elite workforce capable of working in a fast-paced world and ensuring progress in science and technology
École des Ponts ParisTech	no	At the heart of its teaching and research strategy, École des Ponts ParisTech places questions of modelling in mechanical engineering, in materials, in economics, in applied mathematics and environmental sciences, for the civil engineering, transportation, energy and water sectors, and in particular everything relating to urban services, infrastructure management or spatial planning
Osaka Prefecture University	yes	Conventionally, education and research are the two important social missions that universities have to fulfill, but now, regional collaborations and social contributions are strongly sought as the third one, and OPU has been encouraging cooperation among industry, the university and the government as well as regional collaborations ahead of other universities
University of Catania	yes	evaluating and transferring knowledge and technology to the outside world
Pontifical Catholic University of Rio Grande do Sul (PUCRS)	yes	Mission of generating and spreading knowledge, enhancing humanistic and professional formation, which is oriented by quality and relevance to develop a fair and fraternal society
Korea Advanced Institute of Science and Technology (KAIST)	yes	Expansion of cooperation with the regional communities
Cairo University	yes	community services
Ivane Javakhishvili Tbilisi State University	yes	To create, maintain and transfer knowledge
Mahidol University	yes	including analytical and critical thinking, intercultural communication adaptability, information technology, entrepreneurship, and social contribution
Chonbuk National University	no	We will help our students enlarge their thinking, and discover new academic resources, thus they will be our priceless masterpiece proudly representing CBNU
National and Kapodistrian University of Athens	yes	Still perhaps the most prestigious university in the country, the University of Athens has established a tradition in scholarship and constructive participation in the social sphere

University of Erlangen-Nuremberg	yes	The strength of the local connections established by FAU can be seen in the diverse range of co-operations with businesses and research institutes, as well as with educational and cultural institutions throughout the region. FAU constitutes a seedbed for successful business start-ups and enriches cultural diversity
UBC	yes	The University embodies the highest standards of service and stewardship of resources and works within the wider community to enhance societal good
University of Zanjan	no	purpose of educational as well as developmental and physical activities
Belarusian State University	yes	The BSU fulfils its educational, intellectual, cultural and social mission aimed at satisfying the needs and interests of an individual, the society and the nation. The core of the BSU is a union made up of students, post-graduate students, PhD students (hereinafter referred to as learners), the academic staff, researchers and pedagogues which carries out its activities in compliance with the classical traditions of the university education, on the basis of the principles of free academic teaching, researching and disseminating knowledge, on the principles of the combination of centralized state administration with democratic self-government, responsibility for the society, unity of study and research, the link of the science and education with practice
Cardiff University	yes	We aim to demonstrate our commitment to the communities of Cardiff and Wales through knowledge exchange projects that help those who do not traditionally engage with us
London South Bank University	yes	We will create an environment for our staff and students to further develop civic engagement and professional partnerships, and continue to develop pathways into higher education for students from low participation neighbourhoods
Rice University	yes	herefore, as an integral part of the university's mission, we seek a broadly diverse student body where educational diversity increases the intellectual vitality of education, scholarship, service and communal life at Rice
University of Belgrade	yes	Devoted to studying, education, progress and prosperity, the University of Belgrade strives to set the strongest standards in higher education, to cherish and encourage intellectual and personal growth and to stimulate meaningful work and effort which serve to the well-being of the entire society
University of Tunis El Manar	yes	Participate in the development activities of the country, support the various sectors of national activity and prepare students for the creation of projects and economic enterprises - encouraging cultural, sporting and social development
Otto von Guericke University of Magdeburg	yes	The University should take a critical approach towards current developments and make these critical views public
